# Machine learning analysis of the human initiator region reveals key features of different types of core promoters

**DOI:** 10.1101/gad.353623.125

**Published:** 2026-09-01

**Authors:** Torrey E. Rhyne-Carrigg, Long Vo ngoc, Claudia Medrano, Kassidy E. Gillespie, James T. Kadonaga

**Affiliations:** Department of Molecular Biology, University of California, San Diego, La Jolla, California 92093, USA

**Keywords:** transcription, RNA polymerase II, core promoter, initiator, TCT motif, gene expression, humans, *Drosophila*

## Abstract

The initiator (Inr) is the starting point for the transcription of many genes. Here, we generated highly predictive machine learning models of the human Inr region, and determined that the Inr is present in ∼60% of focused human promoters, identified a novel TATA-specific Inr, and detected the overlapping but functionally distinct TCT motif. Quantitative genome-wide analyses revealed a strict and synergistic interaction between the Inr and DPR, an inverse relationship between the TATA and DPR, a flexible and sometimes independent function of the TATA box in relation to the Inr, and different properties of the TCT motif in humans versus *Drosophila*.

## Abstract (precis)

In this study, Rhyne-Carrigg et al. use predictive machine learning to characterize the Initiator (Inr) element, which directs transcription initiation in focused, human gene promoters. They further describe the functional properties of an identified TATA-specific Inr and distinct TCT motifs, illustrating how specific molecular interactions between core promoter elements can dictate transcription regulation.

The RNA polymerase II transcription system is responsible for the expression of protein-coding genes as well as many noncoding RNAs in eukaryotes. The initiation of RNA polymerase II transcription is mediated by the core promoter, which is the stretch of DNA that is approximately −40 to +40 nt relative to the +1 transcription start site (TSS) (for reviews, see Smale and Kadonaga 2003; Vo ngoc et al. 2017a; Haberle and Stark 2018; Roeder 2019; Schier and Taatjes 2020; Sloutskin et al. 2021; Archuleta et al. 2024). The core promoter, often referred to as the “gateway to transcription,” is at a key strategic position in the transcription process, as it is the site of convergence of the signals that lead to initiation.

Core promoters exhibit different TSS patterns, which are termed focused and dispersed (for reviews, see Vo ngoc et al. 2017a; Haberle and Stark 2018; Sloutskin et al. 2021). In focused promoters, transcription initiates at a single site or in a narrow cluster of sites (e.g., ±2 nt relative to the primary site), whereas in dispersed promoters, there are multiple weak TSSs over an ∼50–100 nt region. There is a general association of focused promoters with regulated genes and dispersed promoters with constitutive genes (e.g., see Kadonaga 2012; Serebreni et al. 2023).

Importantly, core promoters do not all function by the same mechanism, but rather, different core promoters can have different activities that are determined by the presence or absence of DNA sequence elements such as the TATA box, downstream promoter region (DPR), initiator (Inr), and TCT motif (Supplemental Fig. S1). (The DPR is a unified version of the MTE and DPE elements [Vo ngoc et al. 2020]. We use the term DPR when referring to the DPE or DPR.) In focused human promoters, the estimated abundances of the TATA, Inr, and DPR are 15%–23%, 40%–56%, and 25%–34%, respectively (Vo ngoc et al. 2017b, 2020). There are no universal core promoter elements, and many human promoters lack any of the known elements. Importantly, the individual core promoter elements confer specific transcriptional regulatory properties. For instance, in *Drosophila*, TATA-specific and DPR-specific enhancers have been identified (Butler and Kadonaga 2001; Juven-Gershon et al. 2008).

Here, we examine the Inr motif, which directs the site of transcription initiation (Smale and Baltimore 1989) and is associated with focused promoters (Kadonaga 2012; Serebreni et al. 2023). In an early analysis of eukaryotic promoters, a YYCA_+1_YYYYY consensus was identified at the TSS (Corden et al. 1980). This motif was incisively demonstrated to be a core promoter element and termed the Inr by Smale and Baltimore (1989), who showed that the Inr by itself can direct accurate transcription initiation. Consistent with this property, a population-scale analysis in *Drosophila* found that genetic variants that create an Inr motif result in stronger and more focused promoters, whereas the disruption of an Inr motif has the opposite effect (Schor et al. 2017). The Inr is recognized by the TFIID complex (Kaufmann and Smale 1994; Purnell et al. 1994; Verrijzer et al. 1995), which also binds to the TATA box and DPR. It is important to note that the Inr in metazoans is different from the TSS sequence in *Saccharomyces cerevisiae*, in which transcription is widely studied (Zhu et al. 2024). In this work, we investigate the ability of the human Inr to direct transcription initiation in focused core promoters.

In our previous studies of the core promoter, we used a combination of high-throughput analysis of randomized promoter elements (HARPE) and machine learning (support vector regression [SVR]) to generate SVR models of the human TATA box and the human and *Drosophila* DPR (Vo ngoc et al. 2020, 2023). These SVR models provide objective, quantitative, data-based predictions of the activity of core promoter motifs and have been found to be more effective at predicting transcriptional activity than consensus sequence-based methods (Vo ngoc et al. 2020). One key advantage of the HARPE-SVR approach is that it involves the analysis of several hundred thousand different DNA sequence variants, which provide much more information than sequences from ∼10,000 to 30,000 natural promoters. In addition, in each HARPE assay, we vary the DNA sequences in only the target region of interest in an otherwise constant promoter backbone. This approach provides high-quality information on a specific segment of the promoter. Thus, in this work, we generated SVR models of the human Inr, which is a key core promoter element that encompasses the TSS (Supplemental Fig. S1). The Inr SVR models, in conjunction with SVR models of the TATA and DPR, revealed key features of different types of core promoters that contain the Inr or the overlapping but functionally distinct TCT motif.

## Results

### SVR models of the human Inr

**Figure 1.**
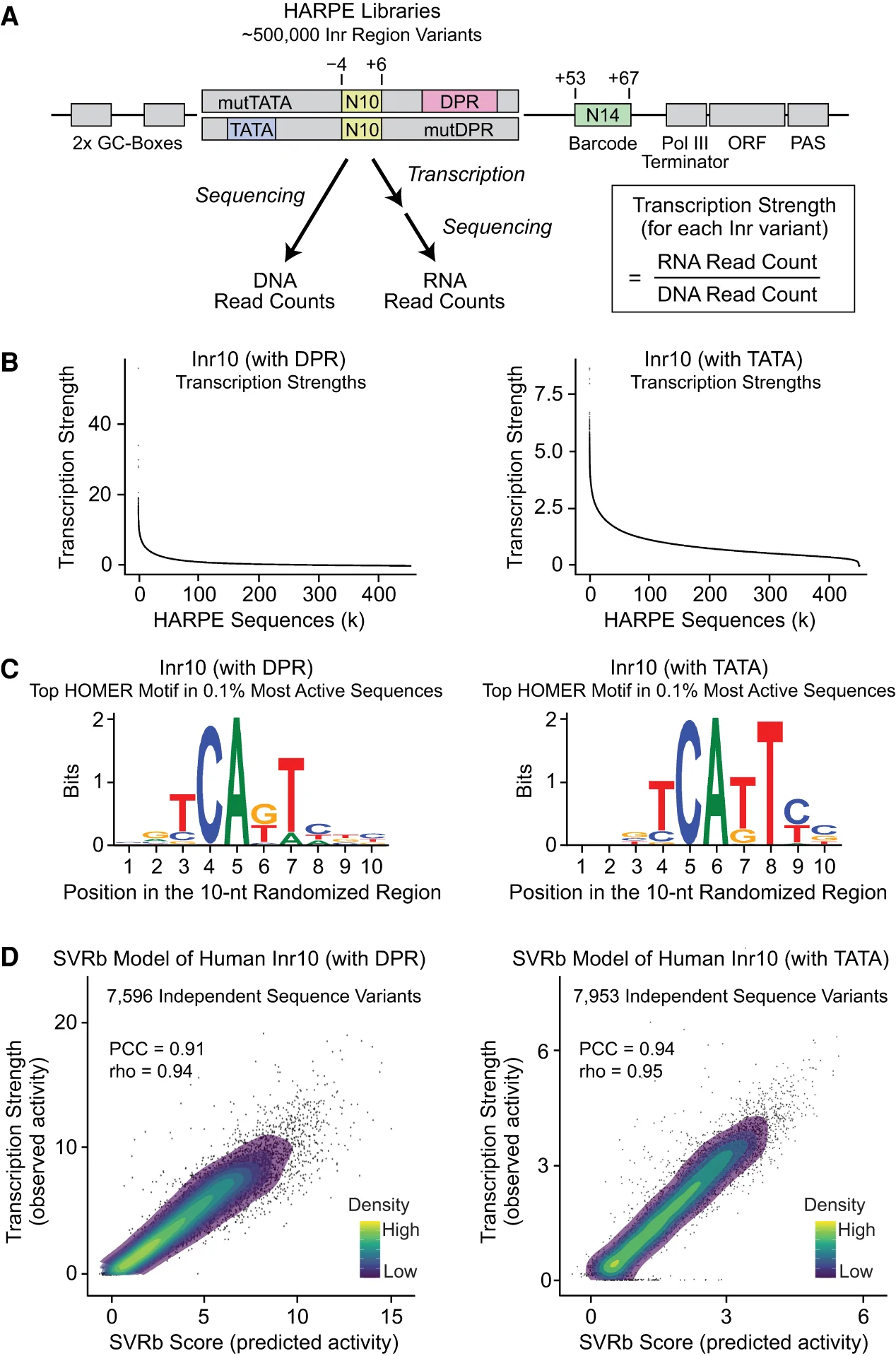
HARPE and SVR analyses of the human Inr in DPR- and TATA-driven promoter backbones. (*A*) Use of the HARPE method for the analysis of the human Inr region. The SCP1-based promoter backbones are identical except for the presence of a DPR (mutTATA) versus a TATA box (mutDPR). The 10 nt region from −4 to +6 (relative to the SCP1 +1 TSS; designated as “Inr10”) was randomized to generate ∼500,000 Inr sequence variants in each promoter backbone. Transcriptional activity was assessed by in vitro transcription with a HeLa nuclear extract. The resulting transcripts were converted to cDNAs, and the downstream transcript-derived barcodes were determined by next-generation sequencing. For each variant, transcription strength was calculated as the ratio of RNA transcript-derived barcode read counts to template DNA-derived barcode read counts. (*B*) Most Inr sequence variants exhibit low transcriptional activity, but a small subset of the variants is highly active. Shown is the transcription strength (with biochemical assays, in order of decreasing activity) of each of the ∼500,000 Inr variants with a DPR (*left*) or TATA box (*right*). (*C*) HOMER analysis of the 0.1% most active Inr variants (in biochemical assays) with a DPR (*left*) or TATA box (*right*) reveals a distinct motif that closely resembles the *Drosophila* Inr. Shown is the web logo of the top HOMER motif. In the DPR-driven promoter, the “A” in the Inr motif is sharply positioned at the fifth nucleotide in the 10 nt randomized region, which corresponds to the A+1 TSS in SCP1, in which the DPR is located from +17 to +35. In the TATA-driven promoter, the “A” in the Inr motif is sharply positioned at the sixth nucleotide in the 10 nt randomized region. For reference, the upstream “T” nucleotide in the SCP1 TATA box is located at the −32 position relative to the peak +1 TSS at the sixth nucleotide. (*D*) The SVRb (trained with biochemical data) models of the human Inr accurately predict the transcription strengths of Inr sequence variants with a DPR (*left*) or TATA box (*right*). Each SVRb model was trained with 200,000 variants and then tested with ∼7000–8000 independent variants (i.e., not used for training) from the HARPE data set. For each of the independent test sequences, the predicted SVRb score was compared with the observed transcription strength. (PCC) Pearson's correlation coefficient, (ρ) Spearman's rank correlation coefficient. SVR scores are relative (i.e., not comparable between models) and are not equivalent to the observed transcription strengths. All panels show average transcription strengths from two independent biological replicates. The SVRb models were trained on the averaged values.

To generate predictive models of the human Inr, we performed HARPE (Fig. 1A) and SVR analyses by the method described by Vo ngoc et al. (2020). To this end, we first measured the biochemical transcription activity of ∼500,000 Inr sequence variants in which a 10 nt region (from −4 to +6 relative to the +1 TSS) in the SCP1 promoter backbone was randomized in a DPR-driven promoter (SCP1 mutTATA) as well as in a separate TATA-driven promoter (SCP1 mutDPR) (Fig. 1A). Because DPR- and TATA-dependent transcription occur by distinct mechanisms (e.g., see Willy et al. 2000; Hsu et al. 2008; Vo ngoc et al. 2020; Serebreni et al. 2023), we analyzed the properties of the Inr in the two different contexts. The HARPE data from independent replicates were found to be reproducible (Supplemental Table S1). A small subset of the sequence variants exhibited high transcriptional activity (Fig. 1B), and HOMER (Heinz et al. 2010) analysis of the top 0.1% of variants revealed a motif that strongly and somewhat surprisingly (in relation to previous human Inr consensus sequences) (e.g., see Vo ngoc et al. 2017b) resembles the *Drosophila* Inr consensus, TCA_+1_KTY or TCA_+1_GTY (Fig. 1C; Supplemental Fig. S2; e.g., see Hultmark et al. 1986; Ohler et al. 2002; FitzGerald et al. 2006). TCA_+1_KTY is, however, the consensus of the most active synthetic Inr sequences and should not be interpreted to be representative of typical Inr elements in natural human promoters.

To provide a generally useful means of evaluating the human Inr, we used the HARPE data to train and optimize SVR models of the human Inr region (Supplemental Fig. S3), and obtained two SVR models, which we termed Inr10 (with DPR) SVRb and Inr10 (with TATA) SVRb. (Inr10 specifies the 10 nt randomized region; [with DPR] and [with TATA] denote the use of DPR- and TATA-driven core promoters, respectively; and SVRb indicates biochemical transcription data.) Importantly, the SVR models provide a numerical score for the predicted activity of any potential Inr sequence. We observed an excellent correlation between the observed transcription strengths of independent (i.e., not used for training) test sequences and their predicted Inr activities (Fig. 1D). Thus, the Inr10 SVRb models of the human Inr provide accurate quantitative predictions of transcriptional activity.

We next investigated whether different libraries with a longer stretch of randomized Inr sequences would yield HARPE data and SVR models that are similar to those obtained with the Inr10 experiments. We therefore constructed and analyzed 14 nt randomized Inr region libraries (from −5 to +9 relative to the SCP1 +1 TSS) and found that the properties of the top 0.1% of variants and the resulting SVR models, termed Inr14 (with DPR) SVRb and Inr14 (with TATA) SVRb (Supplemental Fig. S4), are nearly identical to those obtained with the 10 nt Inr libraries (Supplemental Fig. S5). Therefore, both the Inr10 and Inr14 SVRb models provide reliable predictions for the activity of the human Inr with the DPR as well as the TATA box. We also tested a third library in which both the DPR and the TATA box were mutated in the promoter backbone (SCP1 mutTATA, mutDPR). This library exhibited much lower overall transcription levels but nevertheless yielded the same top HOMER motif (Supplemental Fig. S5; Heinz et al. 2010) and a good SVR model (Supplemental Fig. S4). Thus, the properties of the Inr alone are similar to those of the Inr in combination with a DPR or TATA box.

**Figure 2.**
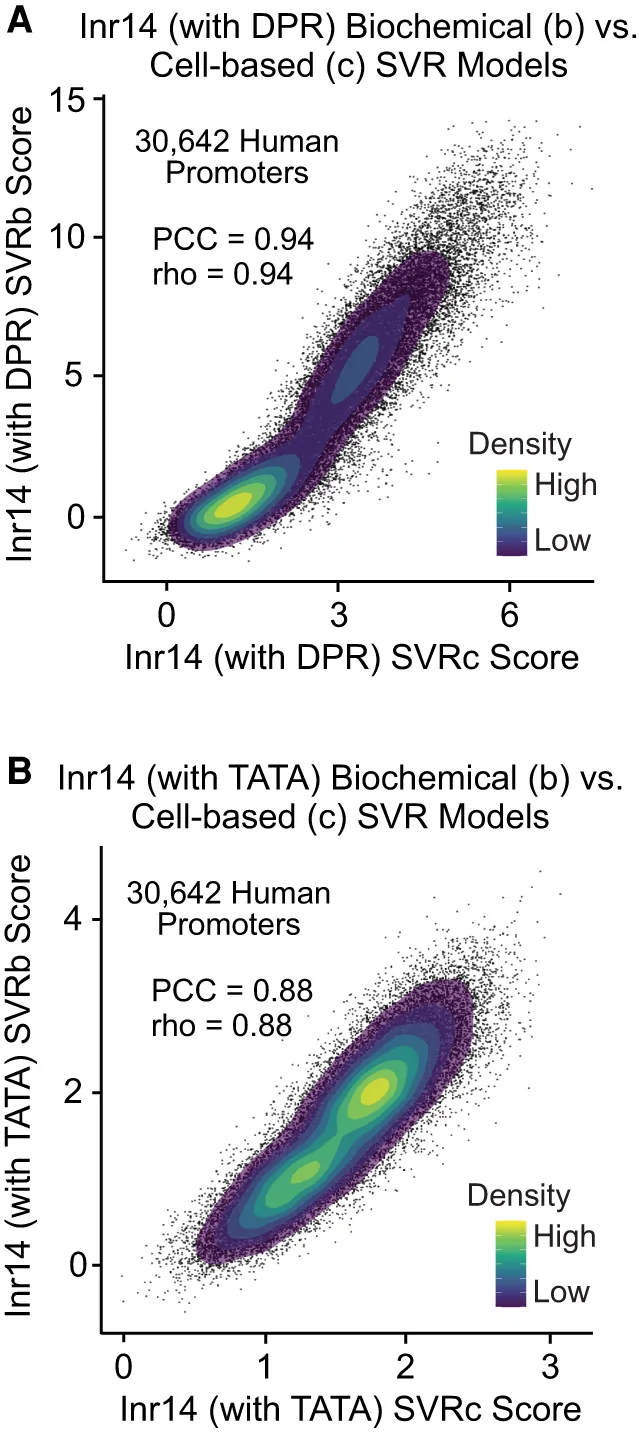
(*A*,*B*) Cell-based (SVRc) models of the human Inr show strong correlation with the corresponding biochemical (SVRb) models. SVR models were generated with biochemical as well as cell-based HARPE data with a 14 nt randomized region (from −5 to +9 relative to the SCP1 +1 TSS; termed “Inr14”) in a DPR-driven (*A*) or TATA-driven (*B*) promoter. SVRc and SVRb scores of 30,642 natural human promoters are compared. TSSs were identified by using GRO-cap data from human GM12878 cells (Core et al. 2014). (PCC) Pearson's correlation coefficient, (ρ) Spearman's rank correlation coefficient.

Third, we tested whether transcription of the Inr region HARPE libraries in cells would yield results that are similar to those obtained in biochemical transcription reactions. We thus performed HARPE analyses by transfection of the 14 nt randomized Inr libraries into HeLa cells. The 0.1% most active cell-transcribed variants exhibited, as did the biochemical experiments (Supplemental Figs. S2, S5), a top HOMER motif that strongly resembles the *Drosophila* Inr (Supplemental Fig. S6). We generated cell-based Inr14 SVRc models (where SVRc indicates a cell-based SVR model; Supplemental Fig. S4) and found that the predictions of the SVRc models strongly correlate with those of their corresponding SVRb models (Fig. 2A,B). Thus, the biochemical and cell-based experiments lead to nearly identical conclusions about the human Inr.

### Analysis of the Inr and TCT motif

**Figure 3.**
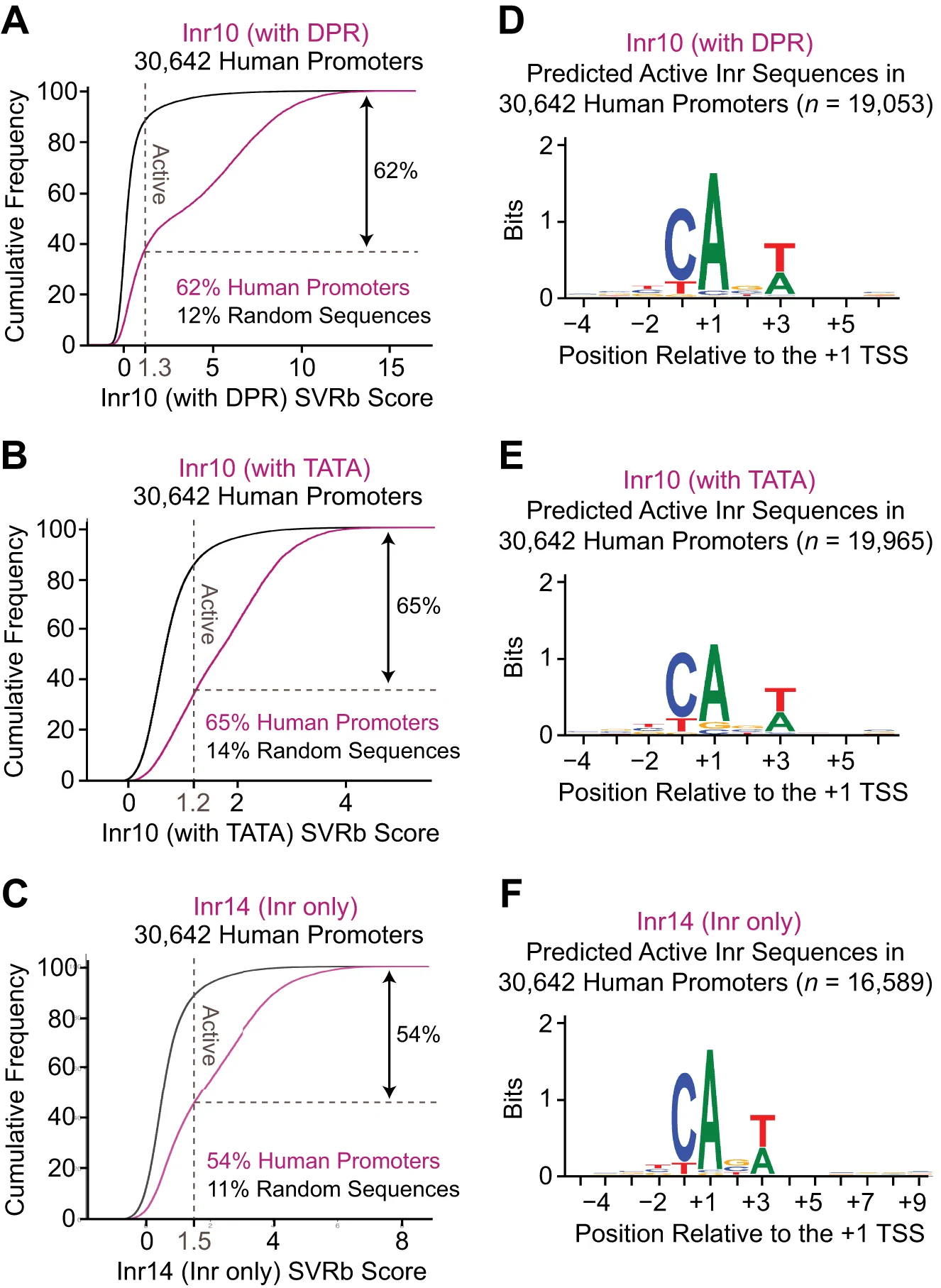
Approximately 60% of human promoters are predicted to contain an active Inr element. (*A–C*) Shown are the cumulative frequencies of Inr SVR scores of sequences in the Inr region (−4 to +6 for Inr10 models; −5 to +9 for Inr14 model) of 30,642 natural promoters in human GM12878 cells. (*A*) With the Inr10 (with DPR) SVRb model, ∼62% of human promoters are predicted to have an active Inr (SVR ≥ 1.3), compared to only ∼12% of 100,000 random 10 nt sequences with the same overall G/C content (61%). (*B*) With the Inr10 (with TATA) SVRb model, ∼65% of human promoters are predicted to have an active Inr (SVR ≥ 1.2), compared to only ∼14% of 100,000 random 10 nt sequences with the same overall G/C content. (*C*) With the Inr14 (Inr only) SVRb model, ∼54% of human promoters are predicted to have an active Inr (SVR ≥ 1.5), compared to only ∼11% of 100,000 random 14 nt sequences with the same overall G/C content. (*D–F*) Active Inr elements have a minimal consensus sequence of CA_+1_NW. Shown are sequence logos of the Inr region of 30,642 natural promoters in human GM12878 cells that are predicted to be active with the Inr10 (with DPR) SVRb model (*D*), Inr10 (with TATA) SVRb model (*E*), or Inr14 (Inr only) SVRb model (*F*). TSSs were identified by using GRO-cap data from human GM12878 cells.

To estimate the abundance of the Inr in natural human promoters, it was first necessary to determine the optimal SVR score thresholds for classifying sequences as active versus inactive. To this end, we carried out a performance assessment for each SVR model to identify the score thresholds at which the models are most accurate (Supplemental Figs. S7–S10). For example, with the Inr10 (with DPR) SVRb model, we found that 10 nt sequences with an SVR score ≥1.3 are predicted to be active as an Inr (Supplemental Fig. S7B). With the SVR score thresholds, we found that ∼60% of natural human focused promoters contain an active Inr (Fig. 3A–C; Supplemental Table S2). This observation is comparable to a recent estimate of ∼40%–56% based on a consensus sequence analysis (Vo ngoc et al. 2017b). In addition, the different SVR models of the Inr (with a DPR, TATA box, or neither element) mostly agree on their predictions of which sequences are active Inr elements (Supplemental Fig. S11). Thus, by using an independent, high-throughput, and function-based approach, these findings provide an improved revised assessment of the high abundance of the Inr in human promoters.

**Figure 4.**
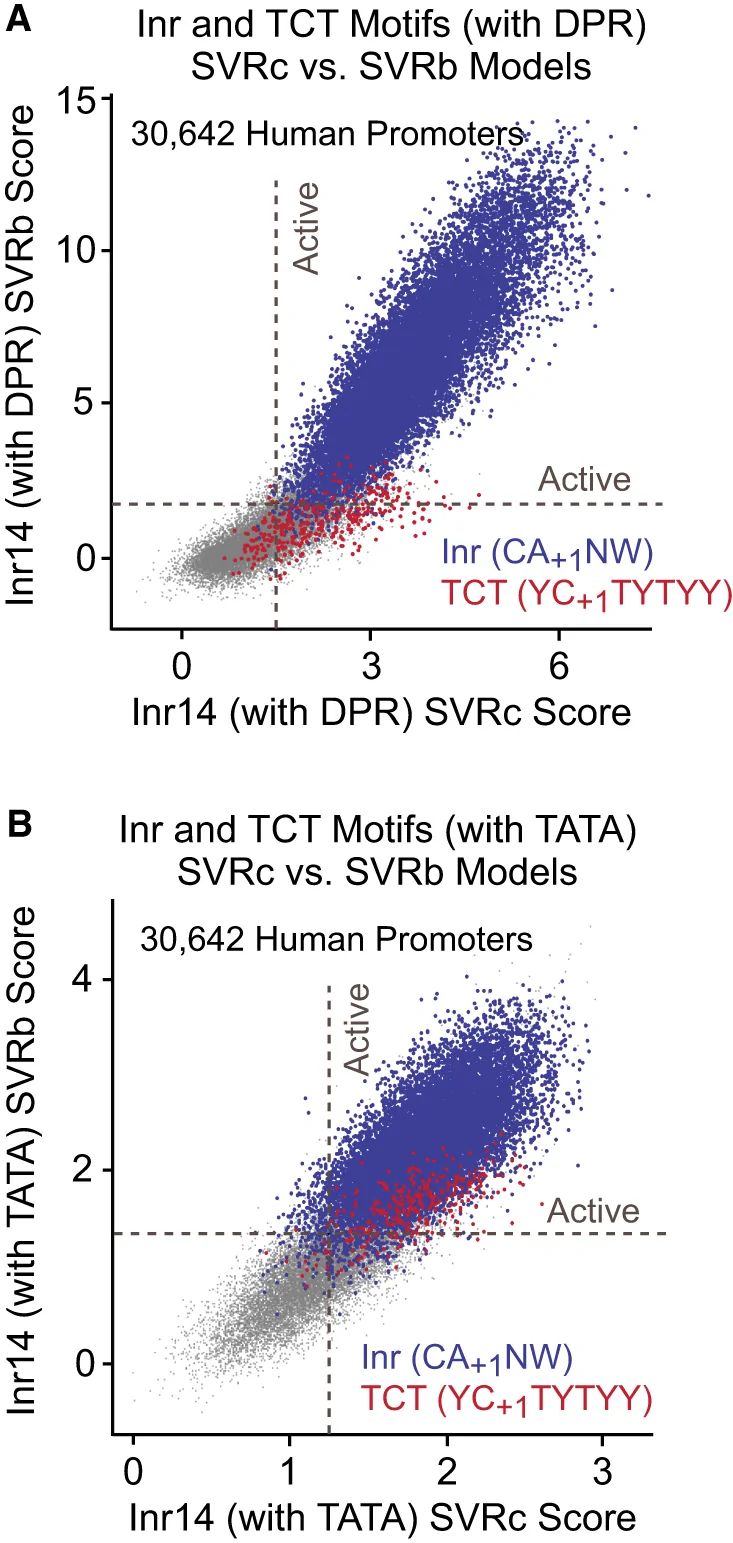
(*A*,*B*) Analysis of the Inr and TCT motifs with biochemical (SVRb) and cell-based (SVRc) models of the human Inr. SVR models were generated with biochemical as well as cell-based HARPE data with a 14 nt randomized region (from −5 to +9 relative to the SCP1 +1 TSS; termed “Inr14”) in a DPR-driven (*A*) or TATA-driven (*B*) promoter. The 14 nt randomized region encompasses both the Inr and TCT motifs. SVRc and SVRb scores are shown for 30,642 natural human focused promoters, with sequences matching the Inr (CA_+1_NW) or TCT (YC_+1_TYTYY) motifs highlighted in blue or red, respectively. TSSs were identified by using GRO-cap data from human GM12878 cells (Core et al. 2014). The red dots are plotted on top of the blue dots for visibility.

We additionally examined the shared features of the predicted active Inr sequences in natural human promoters in GM12878 cells (GRO-cap method) (Core et al. 2014) and K562 cells (csRNA-seq method) (Duttke et al. 2019). To this end, we used the Inr SVRb models to generate sequence logos of all of the predicted active human Inr sequences and identified a minimal CA_+1_NW motif (Fig. 3D–F), which resembles the BBCA_+1_BW consensus that was identified by analysis of natural promoters and estimated to be present in ∼40%–56% of human promoters (Vo ngoc et al. 2017b). The CA_+1_NW consensus thus serves as a minimal Inr reference sequence that is representative of a typical human Inr. Unlike the SVR models, CA_+1_NW does not provide any quantitative information on the transcriptional activity of any Inr that matches this motif. It is useful to note, however, that the Inr SVR models predict that most of the human Inr sequences with a match to the minimal CA_+1_NW Inr motif are active (Fig. 4A,B; Supplemental Fig. S12).

Importantly, in addition to the Inr, the randomized Inr region encompasses the overlapping but functionally distinct TCT motif (Supplemental Fig. S1), which is a rare but biologically important core promoter element that mediates the transcription of ribosomal protein genes and other genes involved in translation in animals (Parry et al. 2010; Wang et al. 2014). We therefore investigated whether the SVR models of the Inr region also recognize the activity of the TCT motif (YC_+1_TYTYY in humans) (Parry et al. 2010). Unlike the Inr, the TCT motif is detected much more distinctly with the Inr14 SVRc models than with the Inr14 SVRb models (Fig. 4A,B; Supplemental Fig. S12). Consistently, as assessed in HARPE assays, TCT sequences are much more active in cells than in biochemical experiments (Supplemental Fig. S13A–D).

These findings prompted us to examine the function of the TCT motif in cells in greater detail. Specifically, we tested whether the higher levels of TCT-derived transcripts in cells (relative to biochemical reactions) might be due to increased transcript stability from a potential 5′-terminal oligopyrimidine (5′TOP) tract, which is a 5′ polypyrimidine stretch in ribosomal protein mRNAs that overlaps with the TCT motif and regulates translation (Gentilella et al. 2017). To investigate this hypothesis, we compared the transcript levels (as assessed in cell-based HARPE functional assays) of promoters that contain RC_+1_YTYTYY versus the TCT consensus of YC_+1_TYTYY. Notably, the upstream R and Y nucleotides at the −1 position are not incorporated into the transcripts. As shown in Supplemental Figure S13, E and F, the RC_+1_YTYTYY promoters are much less active than the YC_+1_YTYTYY promoters. The importance of the Y at position −1 indicates that the TCT element functions independently of the sequence of the mRNA transcript and therefore appears to act primarily at the level of transcription initiation. It is thus likely that the lower levels of TCT motif activity in the biochemical experiments (relative to the cell-based experiments) are due to the depletion or partial inactivation of one or more TCT-specific initiation factors or cofactors in the biochemical extracts.

These studies show that the cell-based HARPE experiments effectively incorporate the activities of both the Inr and TCT motif. Notably, most of the identified TCT motifs are predicted to be active with the SVRc models (Fig. 4A,B; Supplemental Fig. S12). In addition, consistent with previous analyses of the TCT motif (Parry et al. 2010; Wang et al. 2014), a large fraction of the TCT-containing promoters are associated with ribosomal protein genes or genes that encode proteins involved in translation (Supplemental Table S3A,B). Thus, for the analysis of both the Inr and the TCT motif, we recommend the use of the Inr SVRc models. For the study of the Inr only, the Inr10 SVRb models are recommended because they predict Inr activity more accurately than the Inr14 SVRb and Inr14 SVRc models (Fig. 1D; Supplemental Figs. S4, S7).

### An Inr variant that functions specifically with the TATA box

**Figure 5.**
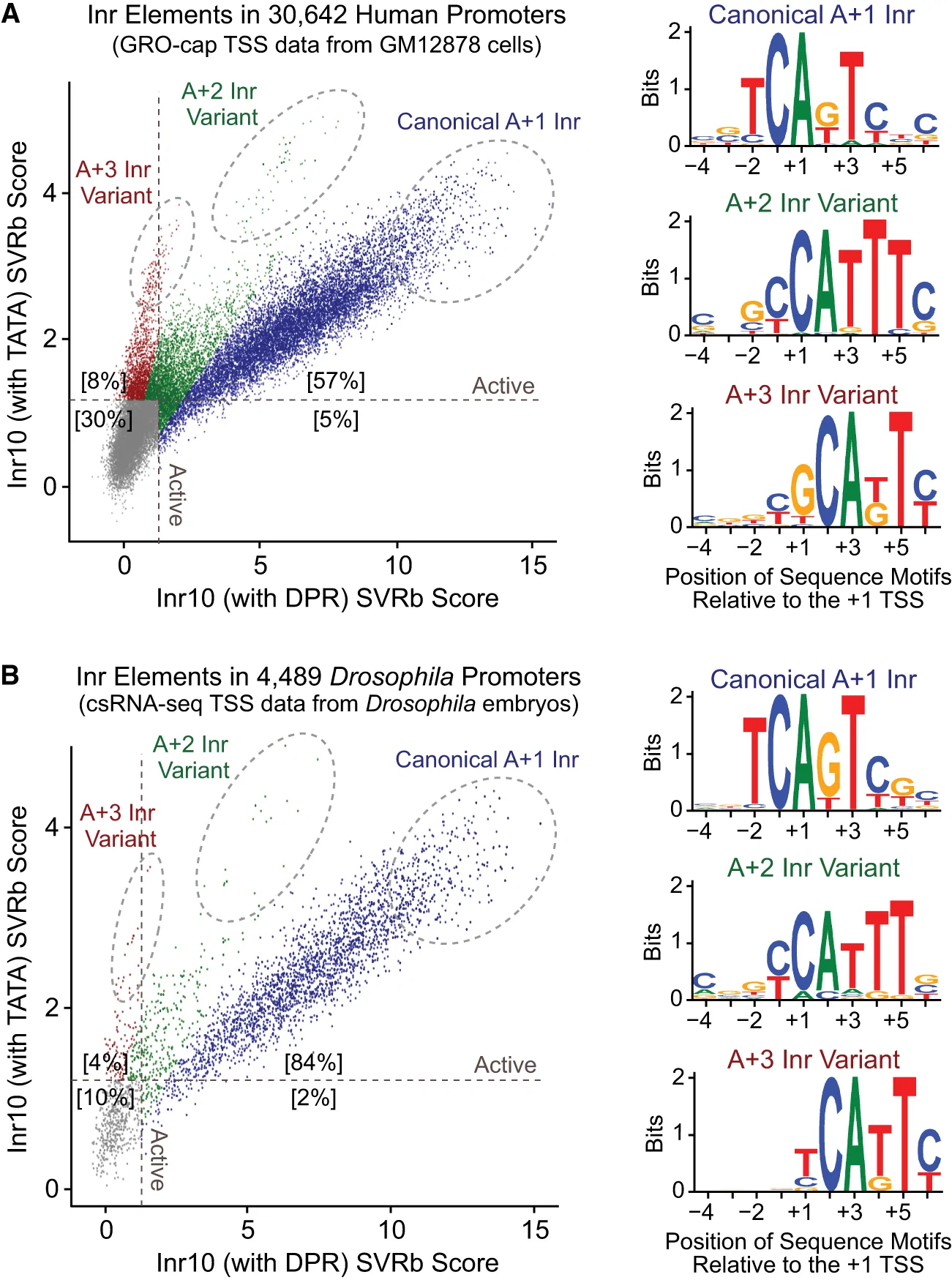
The positioning of the Inr sequence motif relative to the +1 TSS is different in DPR-driven promoters than in TATA-driven promoters. (*A*) Comparison of the predicted Inr activities of 30,642 natural human promoters with the Inr (with DPR) SVR model versus the Inr (with TATA) SVR model reveals three distinct variants, which are labeled according to the position of the A nt in the minimal CAKT Inr motif relative to the observed +1 TSS. The web logos are based on the sequences in the highlighted dashed ovals. (*B*) The three Inr variants are conserved from *Drosophila* to humans. Comparison of the predicted Inr activities of 4489 natural *Drosophila* promoters with the Inr (with DPR) SVR model versus Inr (with TATA) SVR model reveals the same three Inr variants that are seen in human promoters.

We next compared the properties of the Inr in a DPR-driven versus a TATA-driven promoter. By using the Inr (with DPR) and Inr (with TATA) SVRb models, we determined the predicted Inr activities of all of the 1,048,576 possible 10 nt sequences (Supplemental Fig. S14A) as well as 10 million random 14 nt sequences (Supplemental Fig. S14B). This analysis revealed three Inr variants that differ in the positioning of the core Inr CAKT motif within the randomized regions. We then examined the occurrence of the three Inr variants in natural promoters and found that they are conserved from *Drosophila* to humans, as seen in human GM12878 cells (Fig. 5A), human K562 cells (Supplemental Fig. S14C), and *Drosophila* embryos (Fig. 5B). In the natural promoters, the A nucleotide in the core CAKT Inr motif is located at different positions (A+1, A+2, or A+3) relative to the observed +1 TSS. In these analyses, we used data sets that were generated by different methods to ensure that the TSS positions are not biased to any peculiarities of a single method. Hence, the TSSs in the GM12878 cells were identified by the GRO-cap method (Kruesi et al. 2013; Core et al. 2014), and the TSSs in the K562 cells and *Drosophila* embryos were determined with the csRNA-seq method (Duttke et al. 2019).

The predicted activities of these three Inr variants differ in a DPR- versus a TATA-driven promoter (Fig. 5; Supplemental Fig. S14). With the Inr10 (with DPR) SVRb model, the canonical A+1 Inr is most active, the A+2 Inr is moderately active, and the A+3 Inr is inactive or very weakly active. This preference is consistent with the previously observed spacing requirement between the Inr and DPR (Burke and Kadonaga 1997; Kutach and Kadonaga 2000; Vo ngoc et al. 2020, 2023). In contrast, with the Inr10 (with TATA) SVRb model, the A+1, A+2, and A+3 Inr variants are predicted to be of comparable activity. The A+3 variant exhibits distinctly different properties with the TATA box relative to the DPR.

To examine the Inr variants from a different perspective, we compared their occurrence in natural TATA- versus DPR-containing promoters (Supplemental Fig. S15). Strikingly, we found that an active A+3 Inr is present in 8.3% (368/4448) of TATA-containing human promoters, but only in 0.1% (10/7630) of DPR-containing human promoters. Even more notably, an active A+3 Inr is present in 4.6% (46/1009) of TATA-containing *Drosophila* promoters and is completely absent (0/3070) in the DPR-containing *Drosophila* promoters. These findings collectively indicate that the A+3 Inr variant functions specifically with the TATA box relative to the DPR.

### A strong TATA box can drive transcription initiation in the absence of an Inr or DPR

**Figure 6.**
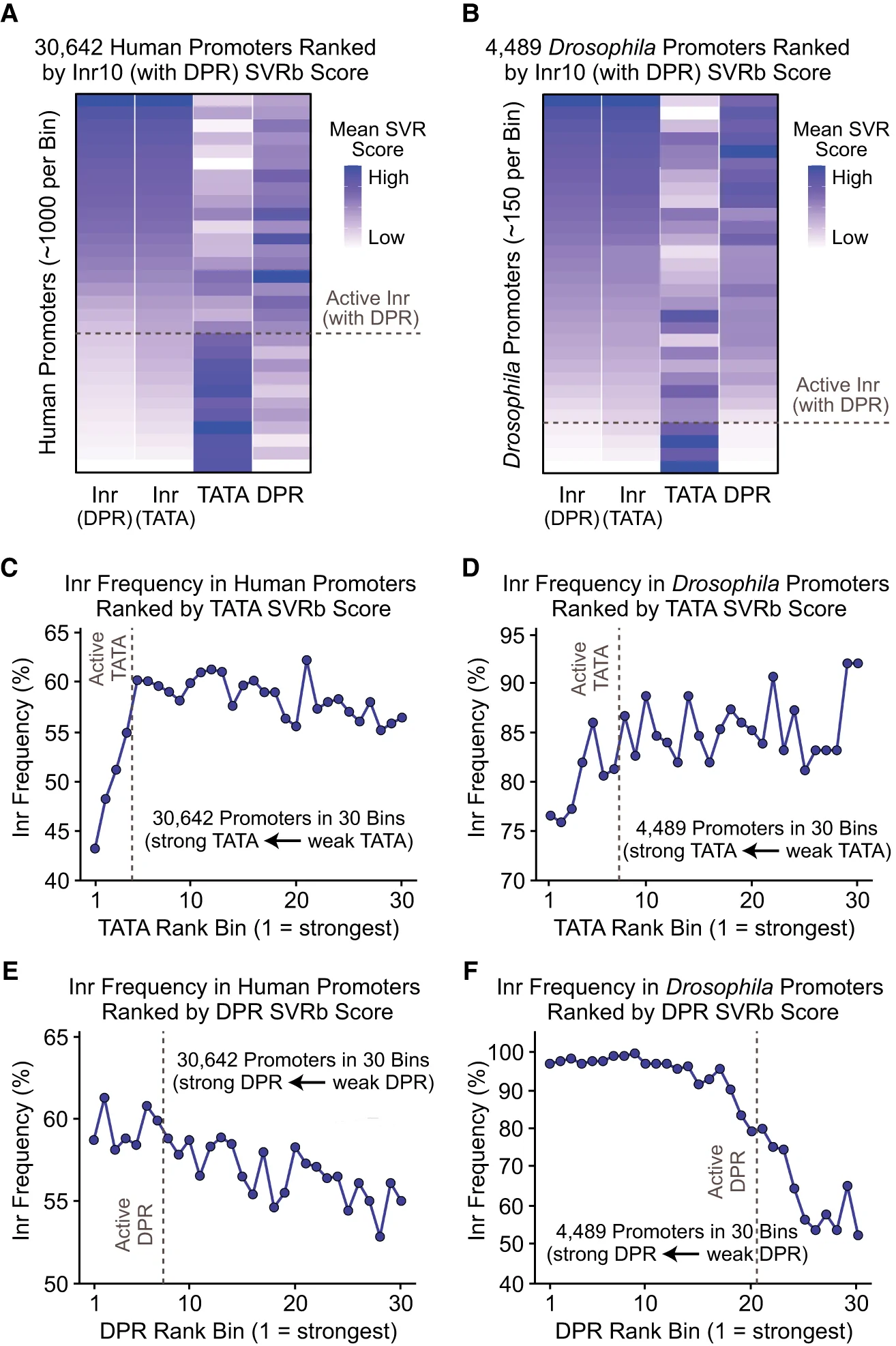
Predicted Inr activity positively correlates with predicted DPR activity and negatively correlates with predicted TATA box activity. (*A–F*) In these analyses, the predicted strengths of core promoter motifs in natural focused promoters from human cells (*A*,*C*,*E*) or *Drosophila* embryos (*B*,*D*,*F*) were assessed by using the human Inr10 (with DPR) SVRb (this study), human Inr10 (with TATA) SVRb (this study), human TATA SVR (Vo ngoc et al. 2020), and human or *Drosophila* DPR SVR (Vo ngoc et al. 2020, 2023) models, where indicated. (*A*,*B*) Positive correlation between the DPR and Inr and negative correlation between the TATA box and Inr in both humans (*A*) and *Drosophila* (*B*). Natural promoters were ranked according to their human Inr10 (with DPR) SVRb scores and divided into 30 bins of approximately equivalent size. Each column shows the mean SVR scores of a core promoter motif (indicated at the *bottom*) in each of the 30 promoter bins. (*C*,*D*) The predicted frequency of active Inr elements sharply declines, particularly in humans, with the predicted strength of active TATA motifs in human (*C*) and *Drosophila* (*D*) promoters. (*E*,*F*) The predicted frequency of active Inr elements increases with the predicted strength of DPR motifs in human (*E*) and *Drosophil*a (*F*) promoters. In *C–F*, active Inr elements were defined to be those that are predicted to be active with both the Inr10 (with DPR) and Inr10 (with TATA) SVRb models. TSSs were identified by using GRO-cap data from human GM12878 cells (Core et al. 2014) (*A*,*C*,*E*) and csRNA-seq data from *Drosophila* embryos (Duttke et al. 2019) (*B*,*D*,*F*).

By using the SVR models of the human Inr in conjunction with our previously generated models of the human TATA box (Vo ngoc et al. 2020), human DPR (Vo ngoc et al. 2020), and *Drosophila* DPR (Vo ngoc et al. 2023), we examined the co-occurrence of these elements in natural human and *Drosophila* promoters. A key feature of these analyses is that they provide quantitative genome-wide assessments rather than anecdotal observations of the relationships between the elements. When the promoters were ranked according to their Inr SVR scores, we observed a striking inverse relationship between predicted Inr and TATA activities (Fig. 6A,B; Supplemental Fig. S16). The inverse relationship between the TATA and DPR (see, e.g., Willy et al. 2000; Hsu et al. 2008; Vo ngoc et al. 2020; Serebreni et al. 2023) can also be clearly seen in the heat maps that are ranked by the DPR SVR scores or the TATA SVR scores (Supplemental Fig. S16).

Further analysis of the Inr and TATA revealed that the predicted frequency of active Inr elements sharply declines, particularly in human promoters, with the predicted strength of active TATA motifs (Fig. 6C,D; Supplemental Fig. S17A,B). This effect was seen in two different human cell lines as well as in *Drosophila* embryos and S2 cells. In contrast, the predicted Inr and DPR activities are positively correlated (Fig. 6A,B; Supplemental Fig. S16), with the predicted frequency of active Inr elements increasing with the predicted strength of DPR motifs (Fig. 6E,F; Supplemental Fig. S17C,D). The positive correlation between the Inr and DPR is consistent with the synergy between these elements (Burke and Kadonaga 1996). A negative correlation between the TATA and Inr was previously observed, as assessed with an Inr consensus sequence (Vo ngoc et al. 2020). The SVR models of the Inr further revealed the sharp decline in Inr frequency with increasing strength of active TATA elements.

To examine the interrelationships between the TATA, Inr, and DPR elements, we carried out permutation testing with natural human and *Drosophila* promoters (Supplemental Fig. S18). This analysis revealed two significantly enriched core promoter types: those containing only a TATA box (TATA^+^, Inr^−^, and DPR^−^) and those containing both an Inr and DPR (TATA^−^, Inr^+^, and DPR^+^). The enrichment of the (TATA^−^, Inr^+^, and DPR^+^) promoters is in accord with the previously noted synergy between the Inr and DPR as well as the co-occurrence of these elements (Kutach and Kadonaga 2000; Vo ngoc et al. 2020). The enrichment of the (TATA^+^, Inr^−^, and DPR^−^) promoters is consistent with the inverse relationships between the TATA and Inr (Fig. 6; Supplemental Figs. S16, S17) and between the TATA and DPR. These findings collectively indicate that the Inr appears to play a more important role in DPR-driven transcription than in TATA-driven transcription and that a strong TATA box can drive transcription initiation in the absence of an Inr or DPR.

It is also notable that we observed a much larger dynamic range of Inr activity with a DPR than with a TATA box (Supplemental Fig. S19). With a DPR-driven promoter, the strongest Inr variants are ∼100-fold more active than the median inactive sequence, whereas with a TATA-driven promoter, the strongest Inr variants are only ∼10-fold more active than the median inactive sequence. This observation is consistent with the co-occurrence and the precise spacing between the Inr and DPR. Moreover, structures of promoter-bound TFIID show the promoter DNA following the surface of TFIID from the Inr through the DPR, whereas there is minimal TFIID contact with the region between the Inr and TATA box (e.g., see Louder et al. 2016). Thus, there appear to be significant differences in the interactions of TFIID with DPR-driven and TATA-driven promoters. The high-throughput HARPE data, the SVR models of the Inr, TATA, and DPR, and the genome-wide human and *Drosophila* promoter analyses collectively support a model in which there is a strict and synergistic interaction between the Inr and DPR, an inverse relationship between the TATA and DPR, and a flexible and sometimes independent function of the TATA box in relation to the Inr.

## Discussion

The Inr is a widely-used transcriptional element that is present in ∼60% of focused human promoters. Here, we combined high-throughput transcriptional analyses with machine learning to generate highly predictive models of the human Inr in different promoter and transcriptional (i.e., biochemical vs. cell-based) contexts. These SVR models, together with our previously generated models of the TATA box and DPR (Vo ngoc et al. 2020, 2023), should be broadly useful for the study of human promoters as well as for applications in synthetic biology. For instance, we used the SVR models of the human and *Drosophila* DPR to identify synthetic DPR sequences with specificity for human transcription factors relative to *Drosophila* factors, and vice versa (Vo ngoc et al. 2023). More broadly, these SVR models could be used to design synthetic promoters with customized functional properties.

The new SVR models are effective at predicting active Inr elements, and it is thus reasonable to ask, anthropomorphically, “What are the SVR models thinking?” To address this question, we first determined the predicted Inr activity of all of the 1,048,576 possible 10 nt sequences and identified the predicted optimal Inr motif (Supplemental Fig. S20A). Second, to evaluate the importance of each position within the Inr region, we randomized the nucleotides at each position and measured the resulting decrease in model performance (Supplemental Fig. S20B). This analysis revealed that the positions corresponding to the minimal CANW Inr sequence are the most important for Inr activity, but also that all 4 nt in this motif contribute to Inr function. Third, we evaluated the effect of every nucleotide substitution at each position across all 10 nt sequences, and identified position- and nucleotide-specific effects on predicted transcriptional activity (Supplemental Fig. S20C). Collectively, these model interpretation analyses uncover the key sequence features that determine Inr activity.

There are different types of core promoters that have different properties based on the presence or absence of motifs such as the TATA box and DPR. For instance, in *Drosophila*, TATA-specific and DPR-specific transcriptional enhancers have been identified (Butler and Kadonaga 2001; Juven-Gershon et al. 2008). The SVR models have enabled us to gain a better understanding of the relationships between the TATA box, Inr, and DPR elements, and have thus provided new insights into the nature of the different types of core promoters.

In this regard, we further investigated the TCT motif. Because the TCT motif is rare and overlaps the Inr, we were not able to generate a TCT-specific SVR model. Therefore, for the analysis of the TCT motif, we used sequences that match the human TCT consensus (YC_+1_TYTYY) and found that the relation between the TATA box and TCT motif in humans is, strikingly, opposite from that seen in *Drosophila* (Supplemental Fig. S21). Specifically, we observed that the TATA box is overrepresented in human TCT promoters and underrepresented in *Drosophila* TCT promoters. We likewise found that the TCT motif is enriched in human TATA-containing promoters and depleted in *Drosophila* TATA-containing promoters. These findings indicate that the properties of the TCT motif are different in humans than in *Drosophila*. This point should be taken into consideration in the analysis of the TCT motif in humans.

It is also important to consider whether different types of core promoter elements are associated with specific biological functions. For instance, the TCT motif is used by the ribosomal protein gene network (Parry et al. 2010). To explore the possible biological functions that may be associated with the two enriched core promoter types (TATA-only and Inr-DPR) (Supplemental Fig. S18), we performed gene ontology (GO) analyses with human promoters (Supplemental Table S4A,B). We found that genes associated with TATA-only promoters are enriched for many functions related to signaling, immune response, and stress response, which suggests that genes that mediate responses to changing conditions may frequently employ TATA-driven transcription. In contrast, the Inr-DPR promoters did not yield any significantly enriched GO terms. These results suggest that there are different biological functions of TATA-only and Inr-DPR core promoters in human gene regulation.

Somewhat remarkably, we found that the optimal human Inr, TCA_+1_KTY, is essentially identical to the *Drosophila* Inr consensus (e.g., see Hultmark et al. 1986; Ohler et al. 2002, FitzGerald et al. 2006). Similarly, in the study by Vo ngoc et al. (2023), we found that the optimal DPR consensus sequence is nearly the same in humans and *Drosophila*. It thus appears that the Inr and DPR motifs have remained largely unchanged over the ∼700 million years of species divergence time between *Drosophila* and humans (Kumar et al. 2022) despite differences in promoter G+C content (Deaton and Bird 2011).

Thus, in conclusion, we were able to generate SVR models of the human Inr and to gain key insights into its occurrence as well as its relation to other core promoter elements at the genome-wide level. There remains, however, much to be learned. In the future, we will seek to identify the DNA motifs and protein factors that drive transcription from the ∼30% of focused human promoters that lack any of the known core promoter elements. We also plan to integrate our understanding of the core promoter with the functions of promoter- and enhancer-binding proteins. In this manner, it may be possible to uncover new unified perspectives into the specific molecular interactions that mediate the regulation of transcription by RNA polymerase II.

## Materials and methods

### HARPE analysis of the Inr

The HARPE plasmid libraries for the Inr were generated as described previously by Vo ngoc et al. (2020). The HARPE vector and SCP1-based promoter backbones, which include constructs with mutations in the DPR and/or the TATA box, are identical to those used previously (Juven-Gershon et al. 2006; Vo ngoc et al. 2020, 2023). For HARPE analysis of the Inr, the 14 nt region from −5 to +9 and the 10 nt region from −4 to +6 (relative to the SCP1 +1 TSS) were randomized to generate ∼500,000 Inr sequence variants within SCP1 promoter backbones containing a DPR (mutTATA), a TATA box (mutDPR), or neither element (mutTATA or mutDPR). In vitro transcription with HeLa nuclear extracts and transfection into HeLa cells were performed as described previously by Vo ngoc et al. (2020). Sample and data processing were performed as described previously by Vo ngoc et al. (2020, 2023). Illumina sequencing of NGS PCR amplicons was performed on NovaSeq 6000 or NovaSeq X Plus sequencing systems at the Institute for Genomic Medicine Genomics Center, University of California, San Diego. Additional information on HARPE library generation, transcription, RNA processing, sequencing, and data analysis is provided in the Supplemental Material.

### Generation and use of the SVR models

Machine learning analyses were performed as described in Vo ngoc et al. (2020, 2023), except that we used functions from the Python package scikit-learn (version 1.3.0) (Pedregosa et al. 2011) instead of the R package e1071 (version 1.7-2). Each SVR model was trained with 200,000 HARPE variants and then evaluated with ∼7000–8000 independent variants (i.e., not used for training). Grid search was used to optimize the hyperparameters cost and γ, and cross-validation was carried out with two independent test sets.

We used SVR models of human Inr (this study), human TATA box (“SVRTATA”; GSE139635) (Vo ngoc et al. 2020), human DPR (“SVRb”; GSE139635 [Vo ngoc et al. 2020], which is the same as “SVRh”; GSE225570 [Vo ngoc et al. 2023]), and *Drosophila* DPR (“SVRd”; GSE225570) (Vo ngoc et al. 2023). We previously provided SVRpredict.R (GSE225570) (Vo ngoc et al. 2023), an R script that facilitates use of the TATA and DPR models. We now provide SVRpredict.py, a Python script analogous to SVRpredict.R, for use with the Inr models. Additional information on model training, optimization, and usage is provided in the Supplemental Material.

### Data availability

The HARPE data, SVRpredict.py script, and SVR models of human Inr have been deposited at Gene Expression Omnibus (accessionno. GSE314877).

## Supplemental Material

Supplement 1
