# Supplementary material for: Machine learning analysis of the human initiator region reveals key features of different types of core promoters
Source: Genes Dev. 2026 Sep 1;40(17-18):1397–407. doi: 10.1101/gad.353623.125 (PMC13532657; doi:10.1101/gad.353623.125)
Supplement: Supplementary file 1 [file Supplemental_Material.pdf]

SUPPLEMENTAL MATERIAL

Machine Learning Analysis of the Human Initiator Region Reveals Key Features of Different Types of Core Promoters

Torrey E. Rhyne-Carrigg, Long Vo ngoc, Claudia Medrano, Kassidy E. Gillespie, and James T. Kadonaga

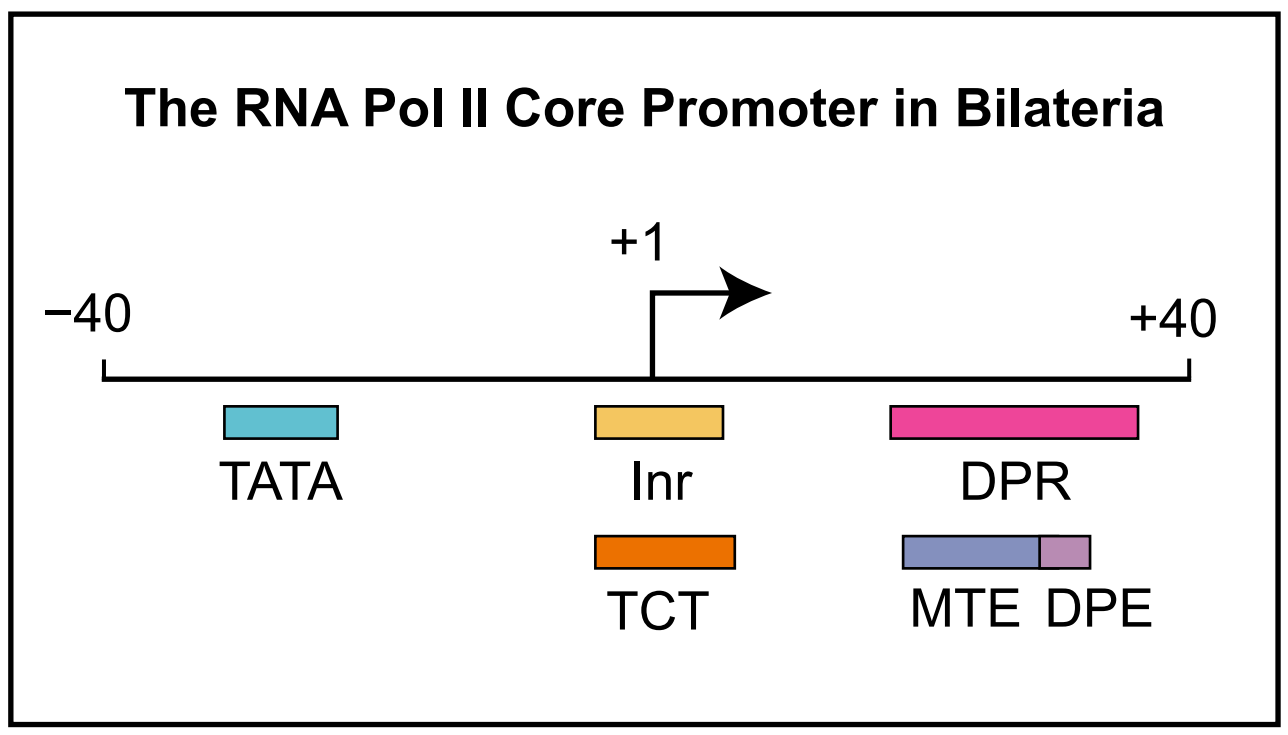

Supplemental Figure S1. The RNA polymerase II core promoter in bilateria. This diagram depicts the DNA sequence motifs that are discussed in the text. The DPR is a unified combination of the MTE and DPE motifs. Because the DPR includes the DPE, we use the term "DPR" when referring to previous work on the DPE or DPR. The Inr and TCT motifs are overlapping but functionally distinct. The drawing is approximately to scale.

Properties of the Top 0.1% Most Active Inr Sequences Observed in the Biochemical Transcription Analysis of Promoters with a 10-nt Randomized Inr Region

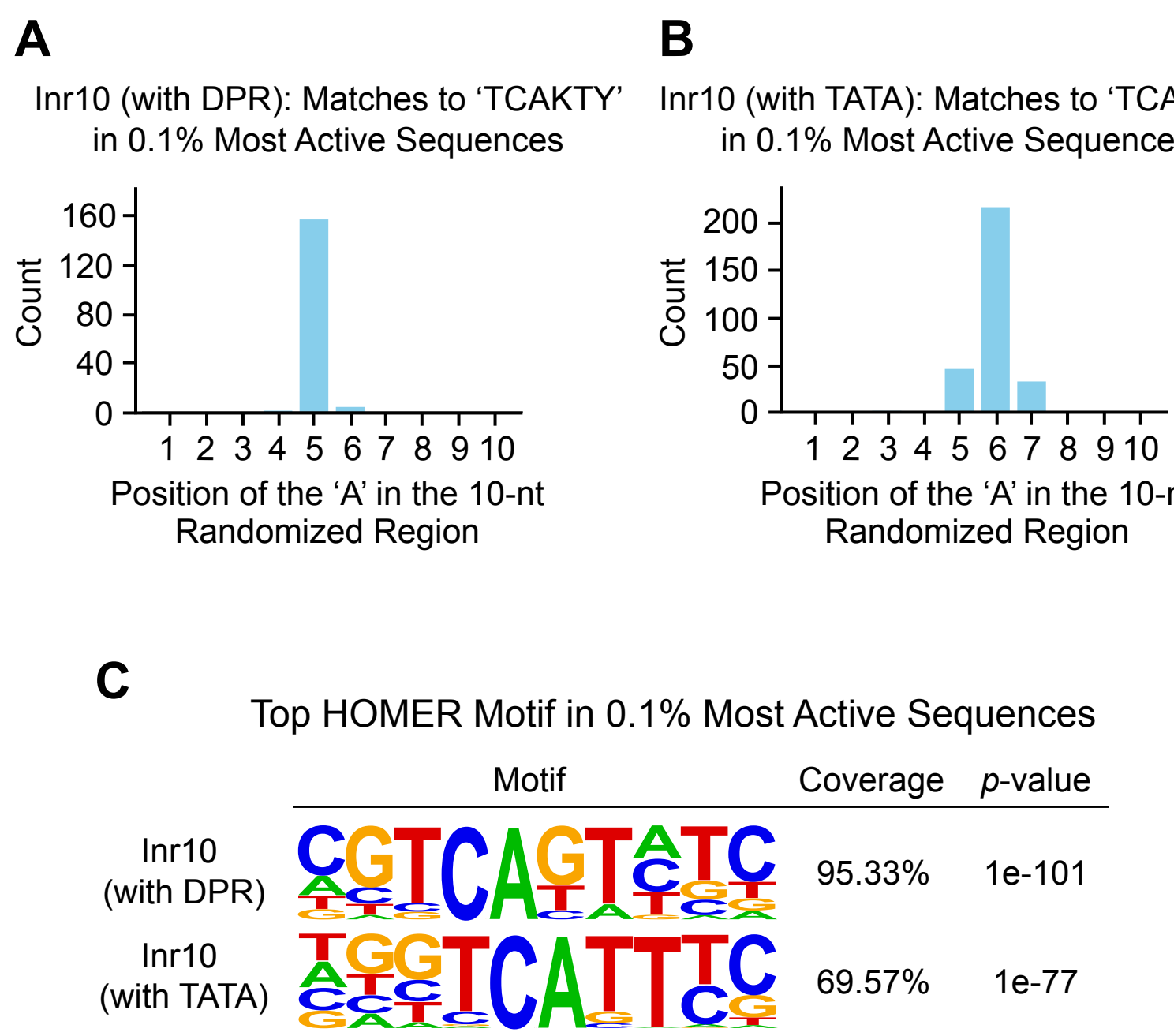

Supplemental Figure S2. Properties of the top 0.1% most active Inr sequences observed in the biochemical transcription analysis of promoters with a 10-nt randomized Inr region. (*A*) In DPR-driven transcription (with a mutant TATA box), the 'A' in the TCAKTY Inr motif is sharply positioned at the 5th nt in the 10-nt randomized region. This position corresponds to the A+1 TSS in SCP1, in which the DPR is located from +17 to +35. This figure depicts the distribution of the 'A' nts in sequences with a perfect match to TCAKTY (n = 162) in the top 0.1% most active Inr (with DPR) sequences (n = 450). (*B*) In TATA-driven transcription (with a mutant DPR), the preferred position of the 'A' in the TCAKTY Inr motif is the 6th nt in the 10-nt randomized region. For reference, the upstream 'T' nucleotide in the SCP1 TATA box is located at the −32 position relative to the peak +1 TSS at the 6th nt. This figure shows the distribution of the 'A' nts in sequences with a perfect match to TCAKTY (n = 293) in the top 0.1% most active Inr (with TATA) sequences (n = 447) (*C*) HOMER analysis of the top 0.1% most active Inr variants with a DPR (top) or TATA box (bottom) reveals a distinct motif that closely resembles the *Drosophila* Inr. Shown are the top HOMER motifs.

Optimization and Cross Validation of the Inr10 (with DPR) SVRb Model

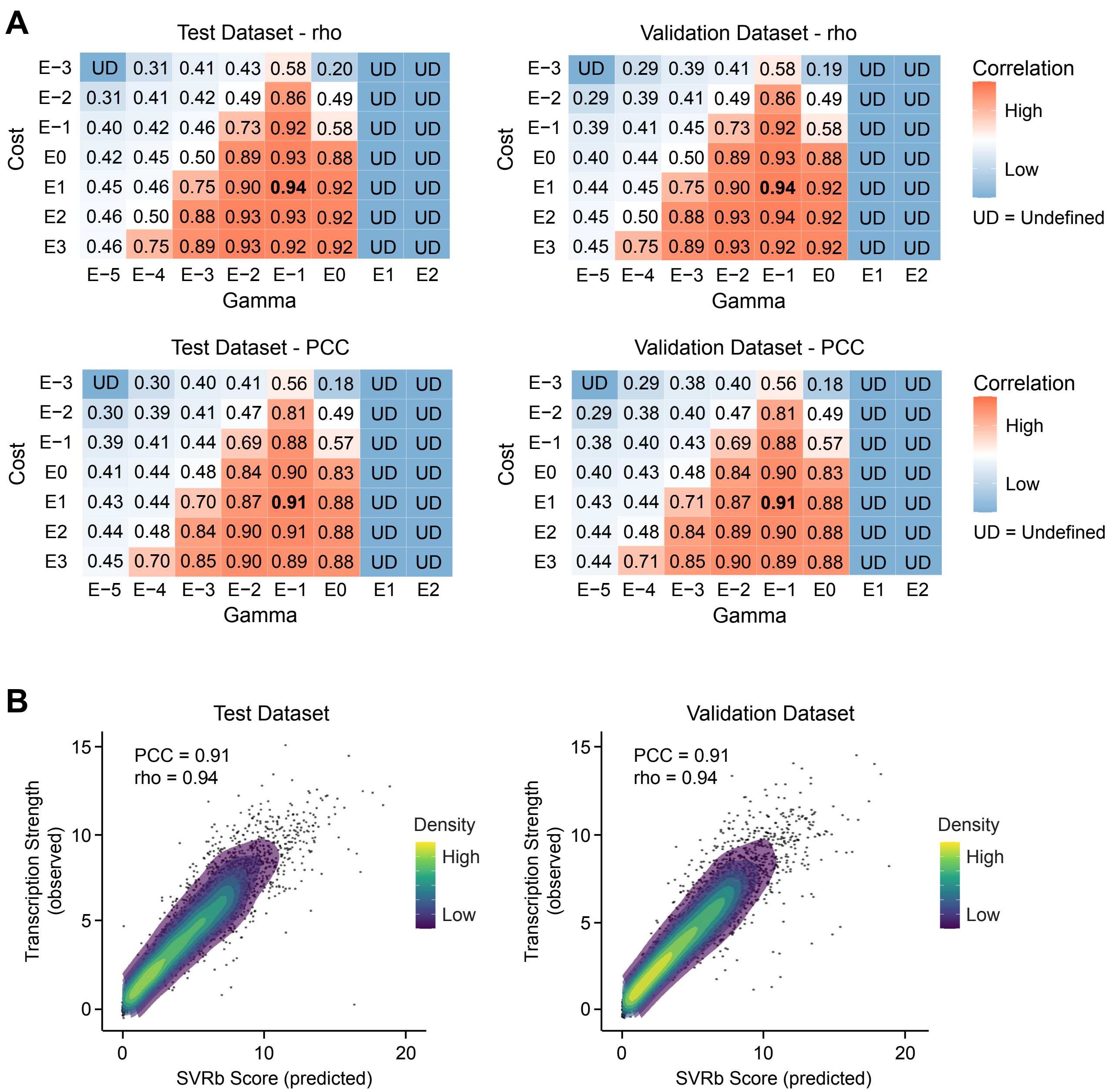

Supplemental Figure S3. Optimization and cross validation of the Inr10 (with DPR) SVRb model. (*A*) Grid search results across different values of the cost and gamma hyperparameters, which control error tolerance and the influence of individual training sequences, respectively. Each matrix displays the values of Spearman's rank correlation coefficient (rho; upper panels) or Pearson's correlation coefficient (PCC; lower panels) for SVR models that were generated with the indicated values of cost and gamma with the test dataset (left panels) or the validation dataset (right panels), which are separate halves of the 7,596 independent test sequences shown in Fig. 1D (left). Undefined (UD) correlation is observed when the prediction of a model is constant regardless of the sequence. The hyperparameter values that were selected for the Inr10 (with DPR) SVR model are cost = 10 = E1 and gamma = 0.1 = E−1. (*B*) Comparison of observed transcription strengths (HARPE data) and predicted transcription strengths [Inr10 (with DPR) SVRb scores] with the test dataset (left) and validation dataset (right). All of the other SVR models were optimized and cross validated in the same manner.

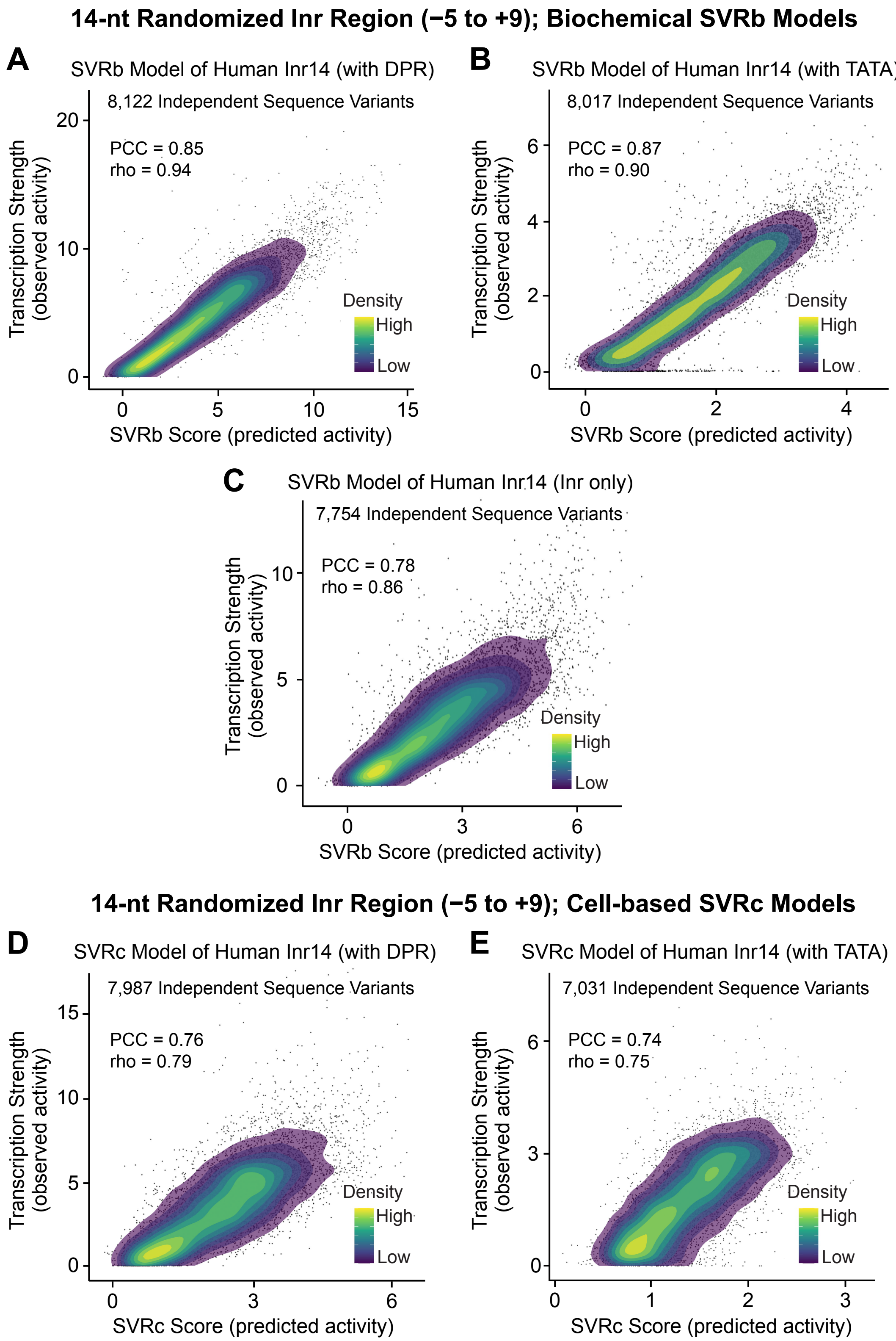

Supplemental Figure S4. Biochemical (SVRb) and cell-based (SVRc) models of the human Inr accurately predict the transcription strengths of Inr sequence variants with a DPR, TATA box, or neither element. SVR models were trained on biochemical (*A,B,C*) or cell-based (*D,E*) HARPE data with a 14-nt randomized Inr region (−5 to +9 relative to the SCP1 +1 TSS; "Inr14") in a DPR-driven (*A,D*), TATA-driven (*B,E*), or Inr-only (*C*) promoter backbone. Each SVR model was trained with 200,000 variants and then tested with approximately 7,000-8,000 independent variants (*i.e.*, not used for training) from the HARPE dataset. For each of the independent test sequences, the predicted SVR score was compared with the observed transcription strength. (PCC) Pearson's correlation coefficient, (rho) Spearman's rank correlation coefficient. SVR scores are relative (*i.e.*, not comparable between models) and are not equivalent to the observed transcription strengths. Shown are the average transcription strengths from two (*A,B,D,E*) or three (*C*) independent biological replicates. The SVR models were trained on the averaged values.

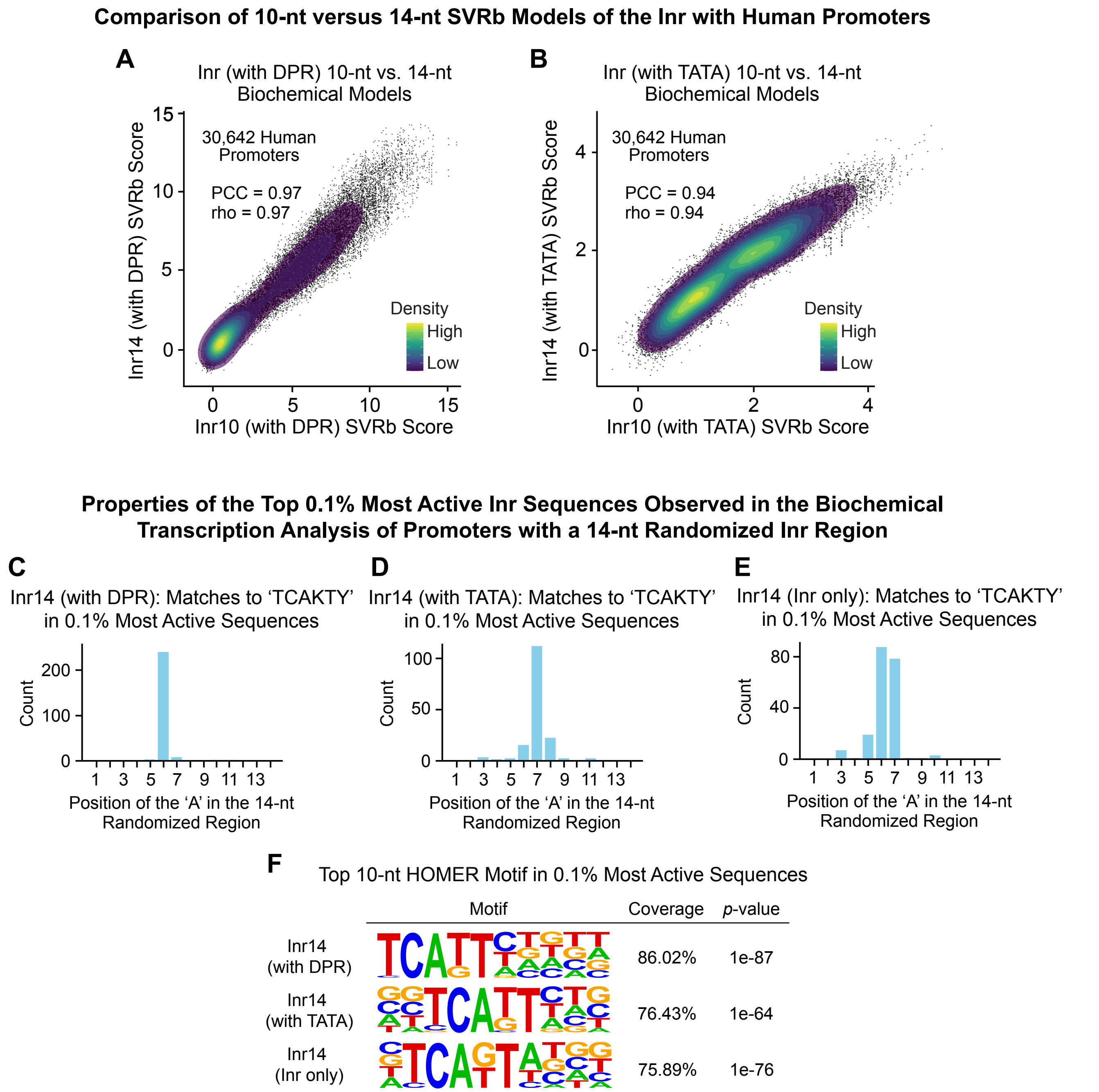

Supplemental Figure S5. Properties of the human Inr based on the biochemical transcription analysis of promoters with a 14-nt randomized Inr region. (*A,B*) Inr14 SVRb models with a DPR-driven promoter (*A*) or a TATA box-driven promoter (*B*) show strong correlation with their corresponding Inr10 SVRb models. Inr10 and Inr14 SVRb scores of 30,642 natural human promoters are compared. (PCC) Pearson's correlation coefficient, (rho) Spearman's rank correlation coefficient. (*C,D,E,F*) Properties of the top 0.1% most active Inr sequences observed in biochemical transcription analyses with a 14-nt randomized Inr region. (*C*) In DPR-driven transcription (with a mutant TATA box), the 'A' in the TCAKTY Inr motif is sharply positioned at the 6th nt in the 14-nt randomized region. This position corresponds to the A+1 TSS in SCP1, in which the DPR is located from +17 to +35. This figure depicts the distribution of the 'A' nts in sequences with a perfect match to TCAKTY (*n* = 251) in the top 0.1% most active Inr (with DPR) sequences (*n* = 465). (*D*) In TATA-driven transcription (with a mutant DPR), the preferred position of the 'A' in the TCAKTY Inr motif is the 7th nt in the 14-nt randomized region. For reference, the upstream 'T' nucleotide in the SCP1 TATA box is located at the −32 position relative to the peak +1 TSS at the 7th nt. This figure shows the distribution of the 'A' nts in sequences with a perfect match to TCAKTY (*n* = 159) in the top 0.1% most active Inr (with TATA) sequences (*n* = 314) (*E*) In the absence of a DPR or a TATA box, the 'A' in the TCAKTY Inr motif is most often positioned at the 6th or 7th nt in the 14-nt randomized region. These positions correspond to the +1 or +2 locations relative to the SCP1 +1 TSS. This figure shows the distribution of the 'A' nts in sequences with a perfect match to TCAKTY (*n* = 197) in the top 0.1% most active Inr (with TATA) sequences (*n* = 336). (*F*) HOMER analysis of the top 0.1% most active Inr variants with a DPR (top), TATA box (middle), or neither (bottom) reveals a distinct motif that closely resembles the *Drosophila* Inr. Shown are the top 10-nt HOMER motifs.

Properties of the Top 0.1% Most Active Inr Sequences Observed in the Cell-based Transcription Analysis of Promoters with a 14-nt Randomized Inr Region

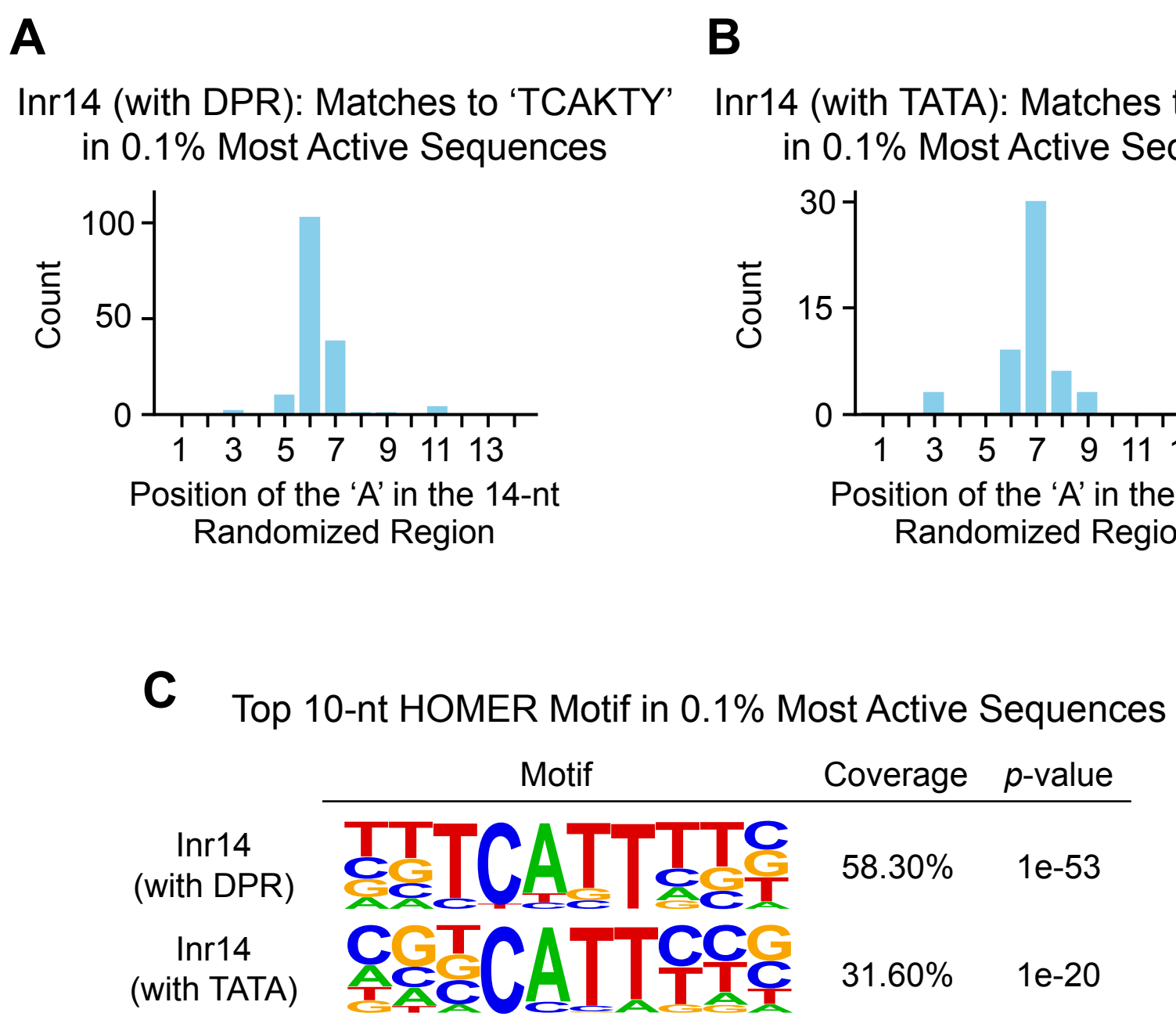

Supplemental Figure S6. Properties of the top 0.1% most active Inr sequences observed in the cell-based transcription analysis of promoters with a 14-nt randomized Inr region. (*A*) In DPR-driven transcription (with a mutant TATA box), the preferred position of the 'A' in the TCAKTY Inr motif is 6th position in the 14-nt randomized region. This position corresponds to the A+1 TSS in SCP1, in which the DPR is located from +17 to +35. This figure depicts the distribution of the 'A' nts in sequences with a perfect match to TCAKTY (n = 158) in the top 0.1% most active Inr (with DPR) sequences (n = 446). (*B*) In TATA-driven transcription (with a mutant DPR), the preferred position of the 'A' in the TCAKTY Inr motif is the 7th nt in the 14-nt randomized region. For reference, the upstream 'T' nucleotide in the SCP1 TATA box is located at the −32 position relative to the peak +1 TSS at the 7th nt. This figure shows the distribution of the 'A' nts in sequences with a perfect match to TCAKTY (n = 51) in the top 0.1% most active Inr (with TATA) sequences (n = 307). (*C*) HOMER analysis of the top 0.1% most active Inr variants with a DPR (top) or TATA box (bottom) reveals a distinct motif that closely resembles the *Drosophila* Inr. Shown are the top 10-nt HOMER motifs.

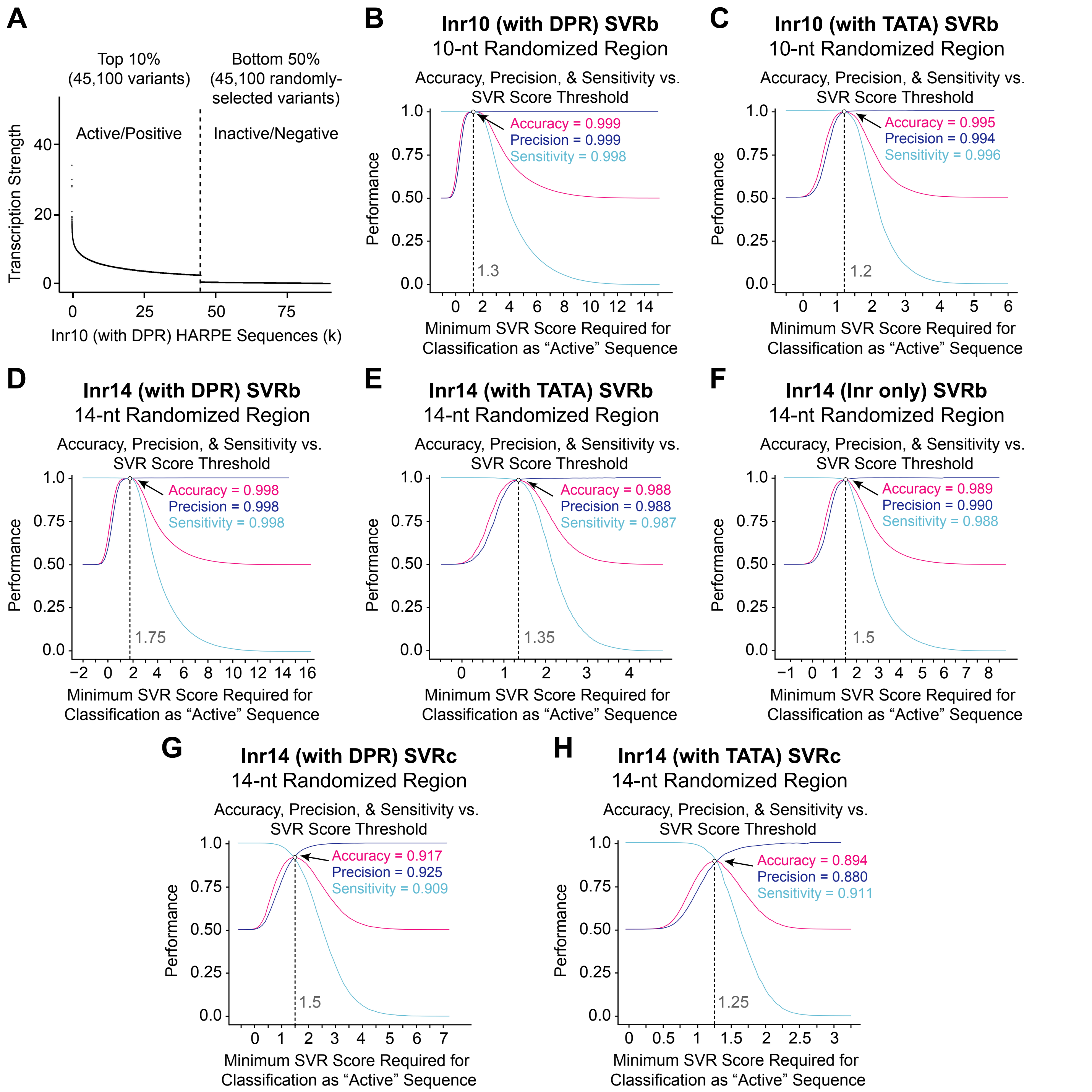

Supplemental Figure S7. Determination of the optimal thresholds for distinguishing active versus inactive Inr elements via SVR performance assessment analyses of the accuracy, precision, and sensitivity versus the minimum SVR scores required for classification as an active sequence. (*A*) Selection of Inr10 (with DPR) HARPE sequence variants for the Inr10 (with DPR) SVRb performance assessment. The top 10% of variants were designated as active/positive for transcription, and an equal number of randomly-sampled sequences in the bottom 50% were designated as inactive/negative. Variants with intermediate activity were excluded. Shown are the transcription strengths (averaged across two independent HARPE replicates) of the selected variants. The same selection criteria were used for the other HARPE experiments. (*B–H*) Performance measures relative to the minimum SVR score required for a positive prediction. Performance was computed by counting true positives (TP), true negatives (TN), false positives (FP), and false negatives (FN). Accuracy [(TP+TN) / (TP+FP+TN+FN)] reflects how often SVR predictions are correct. Precision [TP / (TP + FP)] is the proportion of positive predictions that are correct. Sensitivity (or recall or true positive rate) [TP / (TP + FN)] is the proportion of transcriptionally active variants that are correctly predicted as positives. (*B*) Inr10 (with DPR) SVRb performance is optimal with 1.3 as the threshold for distinguishing active (SVR ≥ 1.3) versus inactive (SVR < 1.3) Inr sequences. (*C*) Inr10 (with TATA) SVRb performance is optimal with 1.2 as the threshold for distinguishing active (SVR ≥ 1.2) versus inactive (SVR < 1.2) Inr sequences. (*D*) Inr14 (with DPR) SVRb performance is optimal with 1.75 as the threshold for distinguishing active (SVR ≥ 1.75) versus inactive (SVR < 1.75) Inr sequences. (*E*) Inr14 (with TATA) SVRb performance is optimal with 1.35 as the threshold for distinguishing active (SVR ≥ 1.35) versus inactive (SVR < 1.35) Inr sequences. (*F*) Inr14 (Inr only) SVRb performance is optimal with 1.5 as the threshold for distinguishing active (SVR ≥ 1.5) versus inactive (SVR < 1.5) Inr sequences. (*G*) Inr14 (with DPR) SVRc performance is optimal with 1.5 as the threshold for distinguishing active (SVR ≥ 1.5) versus inactive (SVR < 1.5) Inr sequences. (*H*) Inr14 (with TATA) SVRc performance is optimal with 1.25 as the threshold for distinguishing active (SVR ≥ 1.25) versus inactive (SVR < 1.25) Inr sequences.

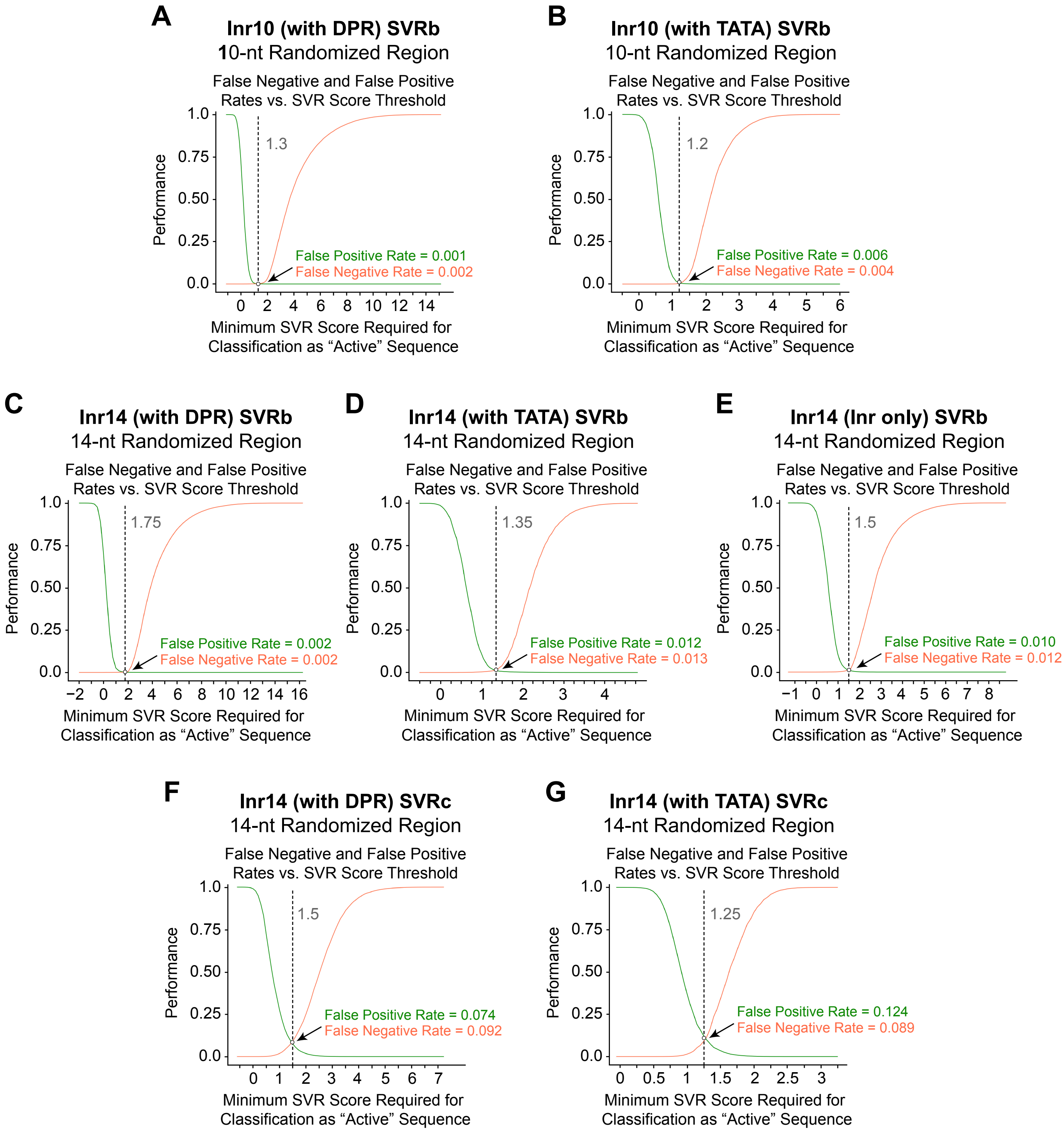

Supplemental Figure S8. Determination of the optimal thresholds for distinguishing active versus inactive Inr elements via SVR performance assessment analyses of the false negative and false positive rates versus the minimum SVR scores required for classification as an active sequence. The HARPE sequence variants used for these performance assessments are the same as in Supplemental Fig. S7. (*A–G*) Performance measures relative to the minimum SVR score required for a positive prediction with the same SVR models described in Supplemental Fig. S7. Performance was computed by counting true positives (TP), true negatives (TN), false positives (FP), and false negatives (FN). False positive rate [FP / (FP + TN)] is the probability for an inactive sequence to be incorrectly predicted as positive. False negative rate [FN / (FN + TP)] = (1 − Sensitivity) is the probability for an active sequence to be incorrectly predicted as negative. The optimal SVR thresholds shown here are the same as those identified in Supplemental Fig. S7.

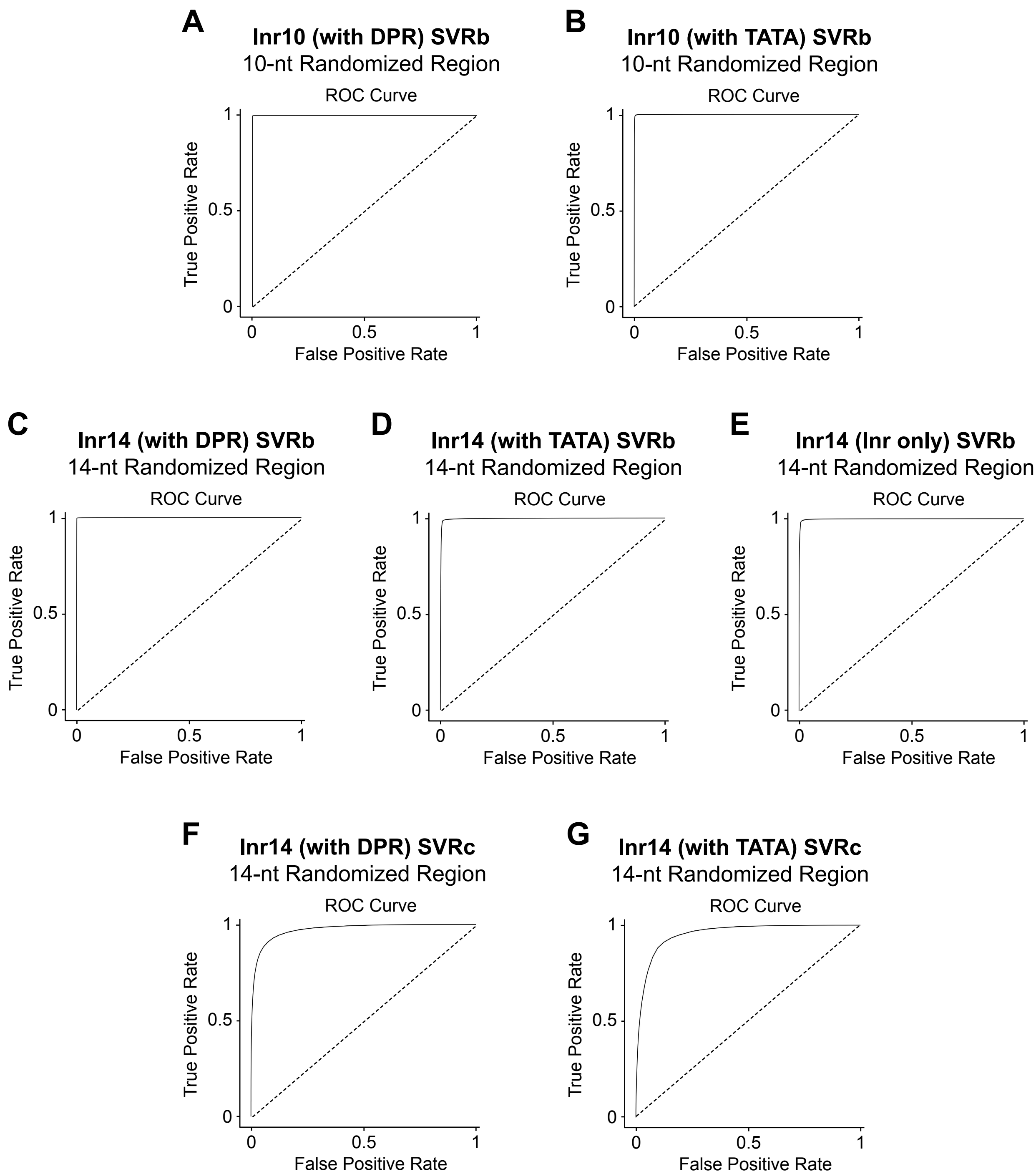

Supplemental Figure S9. Receiver operating characteristic (ROC) curves of the Inr SVR models. The HARPE sequence variants used for the ROC curves are the same as in Supplemental Fig. S7. (*A*–*G*) ROC analysis of the SVR models described in Supplemental Fig. S7. Performance was computed by counting true positives (TP), true negatives (TN), false positives (FP), and false negatives (FN). True positive rate (or sensitivity or recall) [TP / (TP + FN)] is the proportion of transcriptionally active variants that are correctly predicted as positives. False positive rate [FP / (FP + TN)] is the probability for an inactive sequence to be incorrectly predicted as positive.

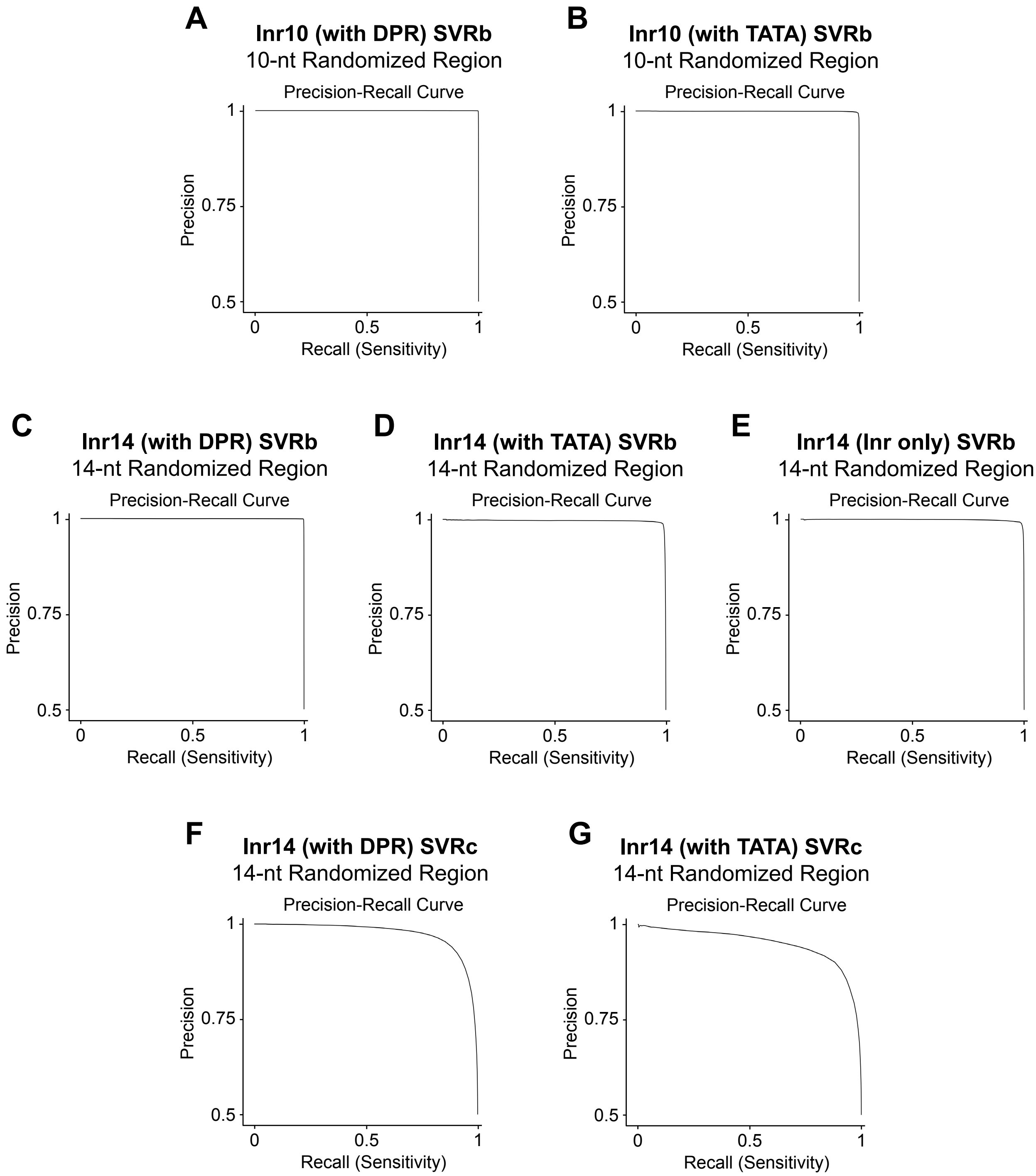

Supplemental Figure S10. Precision-recall (PR) curves of the Inr SVR models. The HARPE sequence variants used for the PR curves are the same as in Supplemental Fig. S7. (*A*–*G*) Precision-recall curves of the SVR models described in Supplemental Fig. S7. Performance was computed by counting true positives (TP), true negatives (TN), false positives (FP), and false negatives (FN). Precision [TP / (TP + FP)] is the proportion of positive predictions that are correct. Recall (or sensitivity or true positive rate) [TP / (TP + FN)] is the proportion of transcriptionally active variants that are correctly predicted as positives.

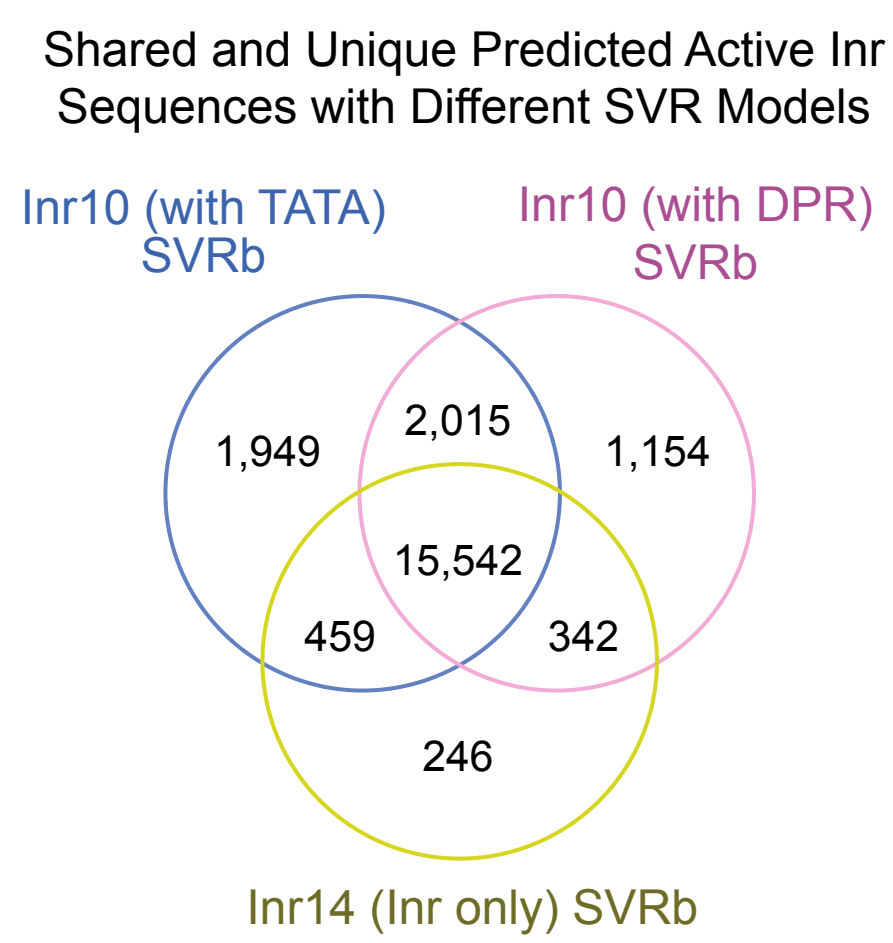

Supplemental Figure S11. The majority of predicted active human Inr sequences are shared across promoter contexts. Shown are the shared and unique predicted active sequences in the Inr region (−4 to +6 for the Inr10 SVR models; −5 to +9 for the Inr14 SVR model) of 30,642 natural human promoters with different SVR models of the human Inr. TSSs were identified by using GRO-cap data from human GM12878 cells (Core et al. 2014).

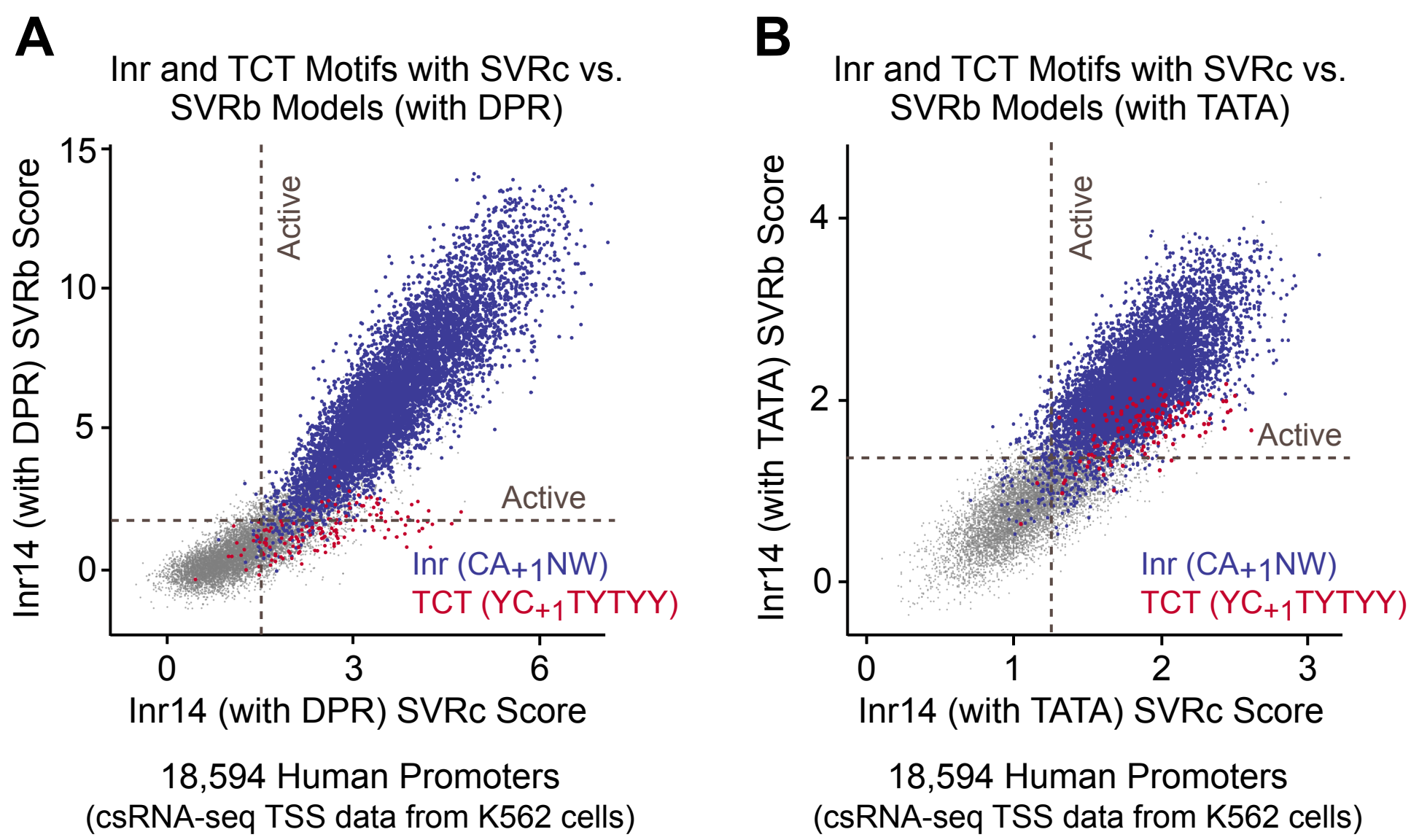

Supplemental Figure S12. The TCT core promoter motif, which overlaps with the Inr motif, can be identified more distinctly with the cell-based Inr 14 SVRc models than with the biochemical Inr14 SVRb models. The Inr14 SVR models were generated with HARPE data obtained from promoters with a 14-nt randomized region (from −5 to +9 relative to the SCP1 +1 TSS). SVR scores are shown for 18,594 natural human promoters, with sequences matching the minimal Inr ($CA_{+1}NW$) or TCT ($YC_{+1}TYTYY$) motifs highlighted in blue or red, respectively. The red dots are plotted on top of the blue dots for visibility. (*A*) Comparison of Inr14 SVR models that were generated with a DPR-driven core promoter. (*B*) Comparison of Inr14 SVR models that were generated with a TATA-driven core promoter.

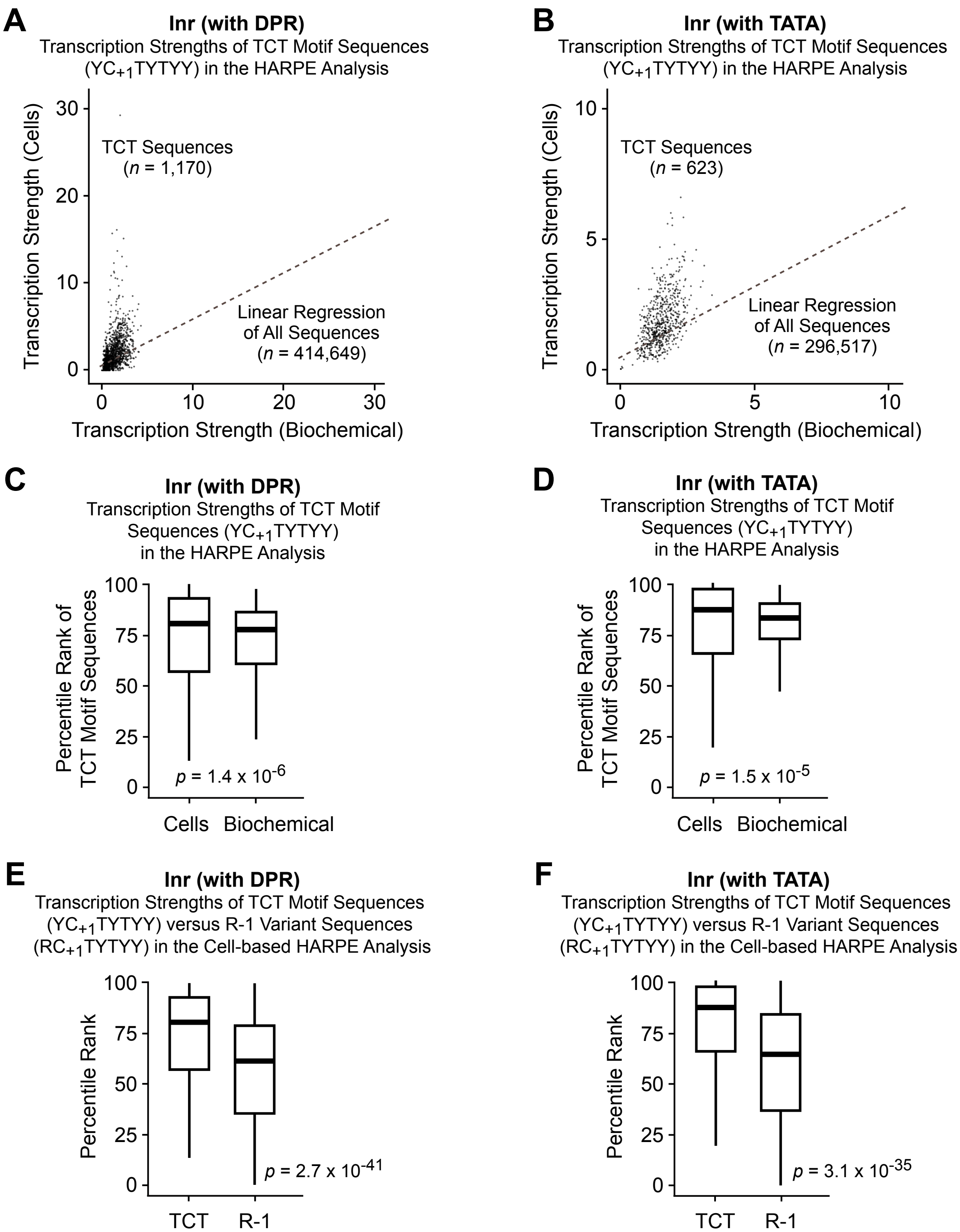

Supplemental Figure S13. HARPE sequence variants that match the TCT motif ($YC_{+1}TYTYY$) exhibit higher transcriptional activity in cells than in vitro. (*A,B*) Observed transcription strengths of TCT motif sequences in the cell-based versus biochemical HARPE experiments with a 14-nt randomized region (−5 to +9) in a DPR-driven (*A*) or TATA-driven (*B*) promoter. The dashed lines show the linear regression of all HARPE sequence variants that were detected in both the cell-based and biochemical experiments. (*C,D*) Percentile ranks of the observed transcription strengths of TCT motif sequences in the cell-based versus biochemical HARPE experiments with a 14-nt randomized region (−5 to +9) in a DPR-driven (*C*) or TATA-driven (*D*) promoter. (*E,F*) Percentile ranks of the observed transcription strengths of TCT motif sequences versus R−1 variant TCT sequences in the cell-based HARPE experiments with a 14-nt randomized region (−5 to +9) in a DPR-driven (*E*) or TATA-driven (*F*) promoter. (*C−F*) The thick horizontal lines are the medians, and the lower and upper hinges are the first and third quartiles, respectively. Whiskers extend from the hinges to the largest or lowest value no further than 1.5 * IQR from the hinge. Data beyond the end of the whiskers (outlying points) are omitted from the box plots. The *p*-values were calculated with a Wilcoxon rank-sum test to compare the percentile distributions.

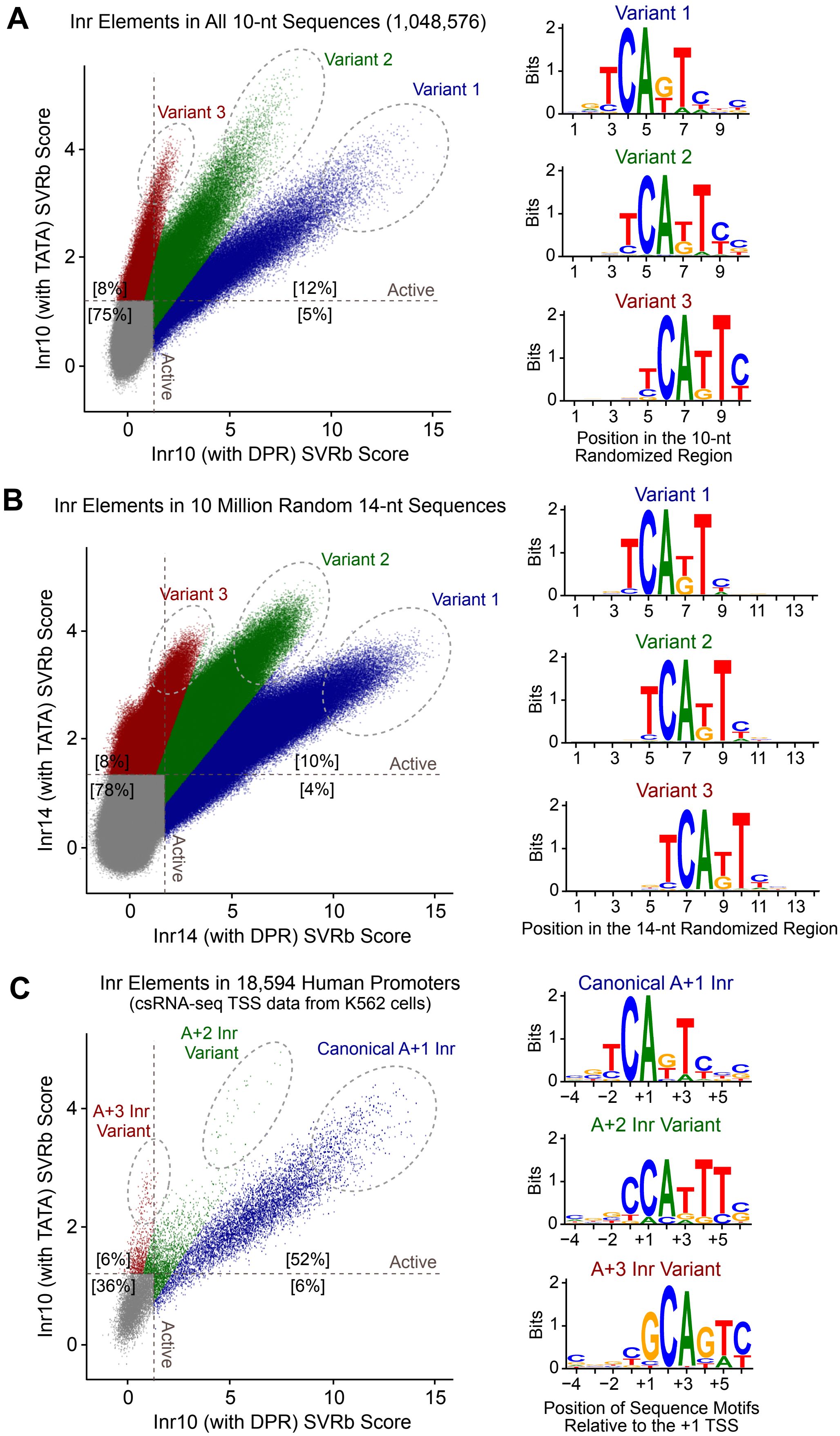

Supplemental Figure S14. The positioning of the Inr sequence motif relative to the +1 TSS is different in DPR-driven promoters than in TATA-driven promoters. (*A,B*) Comparison of the predicted Inr activities of all possible 10-nt sequences (*A*) or 10 million random 14-nt sequences (*B*) with the Inr (with DPR) SVR models versus the Inr (with TATA) SVR models reveals three distinct variants with different positioning of the core Inr CAKT motif within the 10-nt (*A*) or 14-nt (*B*) randomized region. The web logos are based on the sequences in the highlighted dashed ovals. (*C*) Comparison of the predicted Inr activities of 18,594 natural human promoters with the Inr (with DPR) SVR model versus Inr (with TATA) SVR model reveals the same three variants, which are labeled according to the position of the A nt in the core CAKT Inr motif relative to the observed +1 TSS.

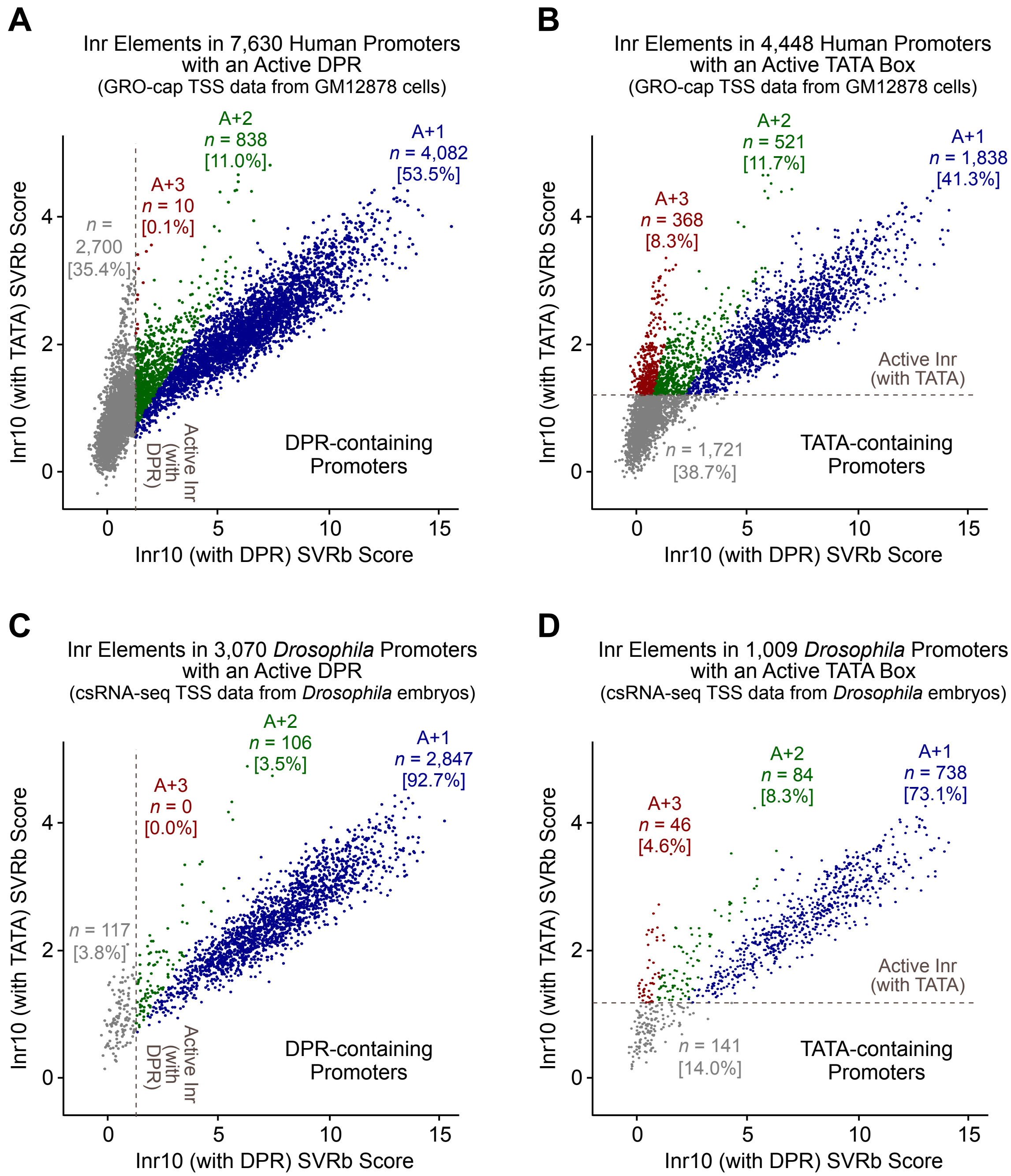

Supplemental Figure S15. The positioning of the Inr sequence motif relative to the +1 TSS is different in natural DPR-containing promoters than in natural TATA-containing promoters. (*A,B*) Comparison of the predicted Inr activities of 7,630 DPR-containing (*A*) and 4,448 TATA-containing (*B*) human promoters with the Inr (with DPR) SVR model versus the Inr (with TATA) SVR models. (*C,D*) Comparison of the predicted Inr activities of 3,070 DPR-containing (*C*) and 1,009 TATA-containing (*D*) *Drosophila* promoters with the Inr (with DPR) SVR model versus the Inr (with TATA) SVR models. DPR-containing promoters were identified by using the SVR models of the human DPR (*A*) and the *Drosophila* DPR (*C*) (Vo ngoc et al. 2020, 2023). (*B,D*) TATA-containing promoters were identified by using the SVR model of the human TATA box (Vo ngoc et al. 2020).

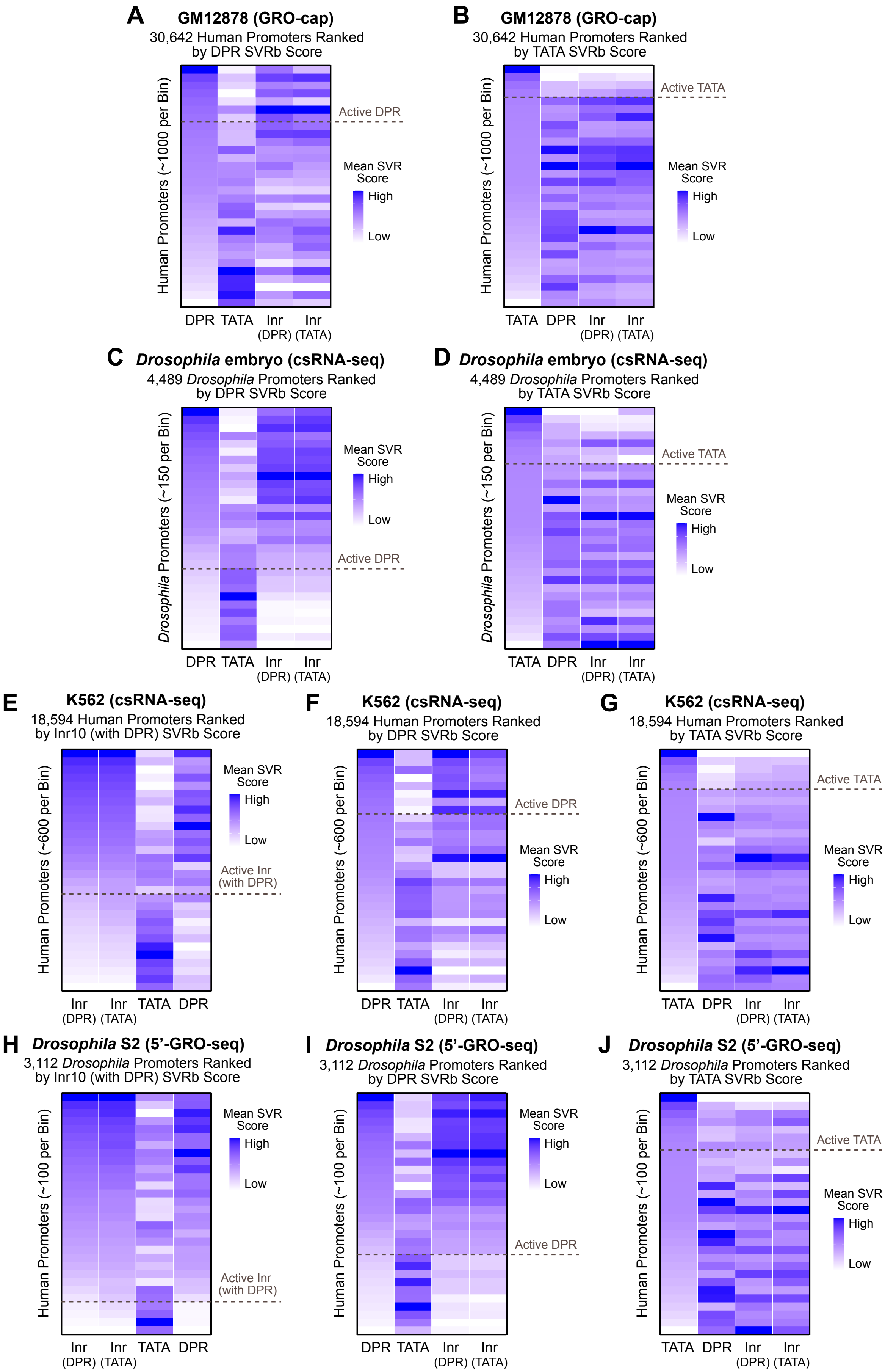

Supplemental Figure S16. Predicted Inr activity positively correlates with predicted DPR activity and negatively correlates with predicted TATA box activity in humans as well as in *Drosophila*. Natural promoters were ranked according to the indicated SVR scores and divided into 30 bins of approximately equivalent size. For each bin, the mean SVR scores for human Inr (with DPR), human Inr (with TATA), human TATA box (Vo ngoc et al. 2020), and human or *Drosophila* DPR (Vo ngoc et al. 2020, 2023) were determined. (*A,B*) Predicted correlations in human GM12878 cells. TSSs were identifed by using GRO-cap data. (*C,D*) Predicted correlations in *Drosophila* embryos. TSSs were identified by using csRNA-seq data. (*E–G*) Predicted correlations in human K562 cells. TSSs were identified by using csRNA-seq data. (*H–J*) Predicted correlations in *Drosophila* S2 cells. TSSs were identified by using 5'-GRO-seq data.

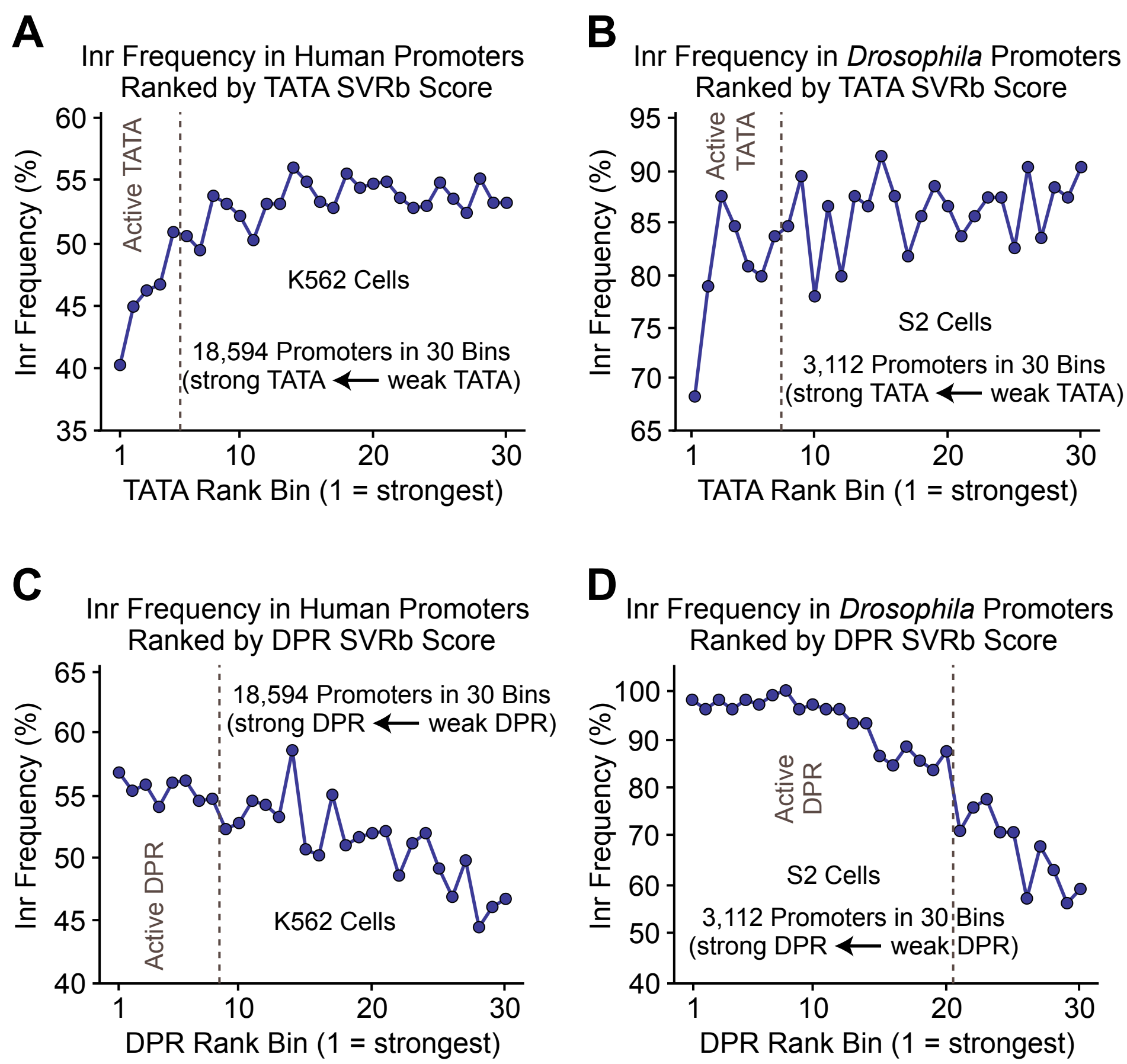

Supplemental Figure S17. The predicted frequency of active Inr elements decreases with the predicted strength of TATA motifs and increases with the predicted strength of DPR motifs in human K562 cells as well as in *Drosophila* S2 cells. Shown are the predicted frequencies of active Inr elements as a function of the predicted strength of the TATA box (*A,B*) or DPR (*C,D*) in human (*A,C*) and *Drosophila* (*B,D*) promoters. Natural promoters were ranked according to their human TATA box scores (Vo ngoc et al. 2020) or human or *Drosophila* DPR scores (Vo ngoc et al. 2020, 2023) and divided into 30 bins of approximately equivalent size. Active Inr elements were defined to be those that are predicted to be active with both the Inr10 (with DPR) and Inr10 (with TATA) SVR models. TSSs were identifed by using csRNA-seq data from human K562 cells (*A,C*) and 5'-GRO-seq data from *Drosophila* S2 cells (*B,D*).

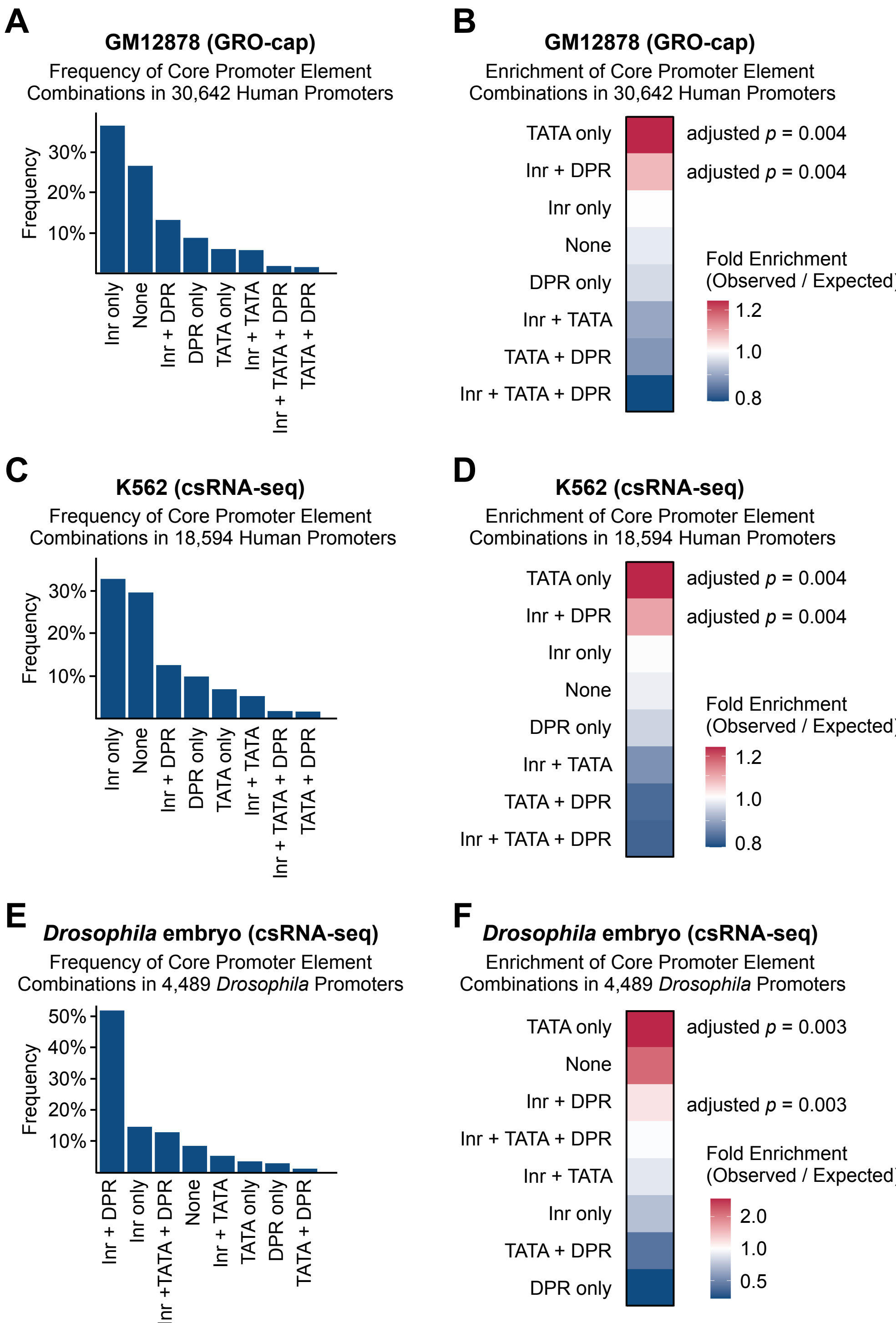

Supplemental Figure S18. Predicted frequency and enrichment of core promoter element combinations in human and *Drosophila* promoters. The predicted frequency of each combination of elements in natural promoters was determined by using the SVRb models of the human Inr, human TATA box (Vo ngoc et al. 2020), and human or *Drosophila* DPR (Vo ngoc et al. 2020, 2023). Active Inr elements were defined to be those that are predicted to be active with both the Inr10 (with DPR) and Inr10 (with TATA) SVR models. The predicted enrichment of each combination of elements was determined by comparing its observed frequency in natural promoters to the expected frequency based on permutation testing (n = 1,000 permutations), where the binary activity value (active or inactive) for each element was shuffled between promoters. The p-values reflect the proportion of permutations with frequencies equal to or greater than observed (0 out of 1,000 for all values shown) and were adjusted for multiple comparisons by using the Benjamini-Hochberg correction. (*A,B*) Core promoter combinations based on TSSs that were identified by using GRO-cap data from human GM12878 cells. (*C,D*) Core promoter combinations based on TSSs that were identified by using csRNA-seq data from human K562 cells. (*E,F*) Core promoter combinations based on TSSs that were identified by using csRNA-seq data from *Drosophila* embryos.

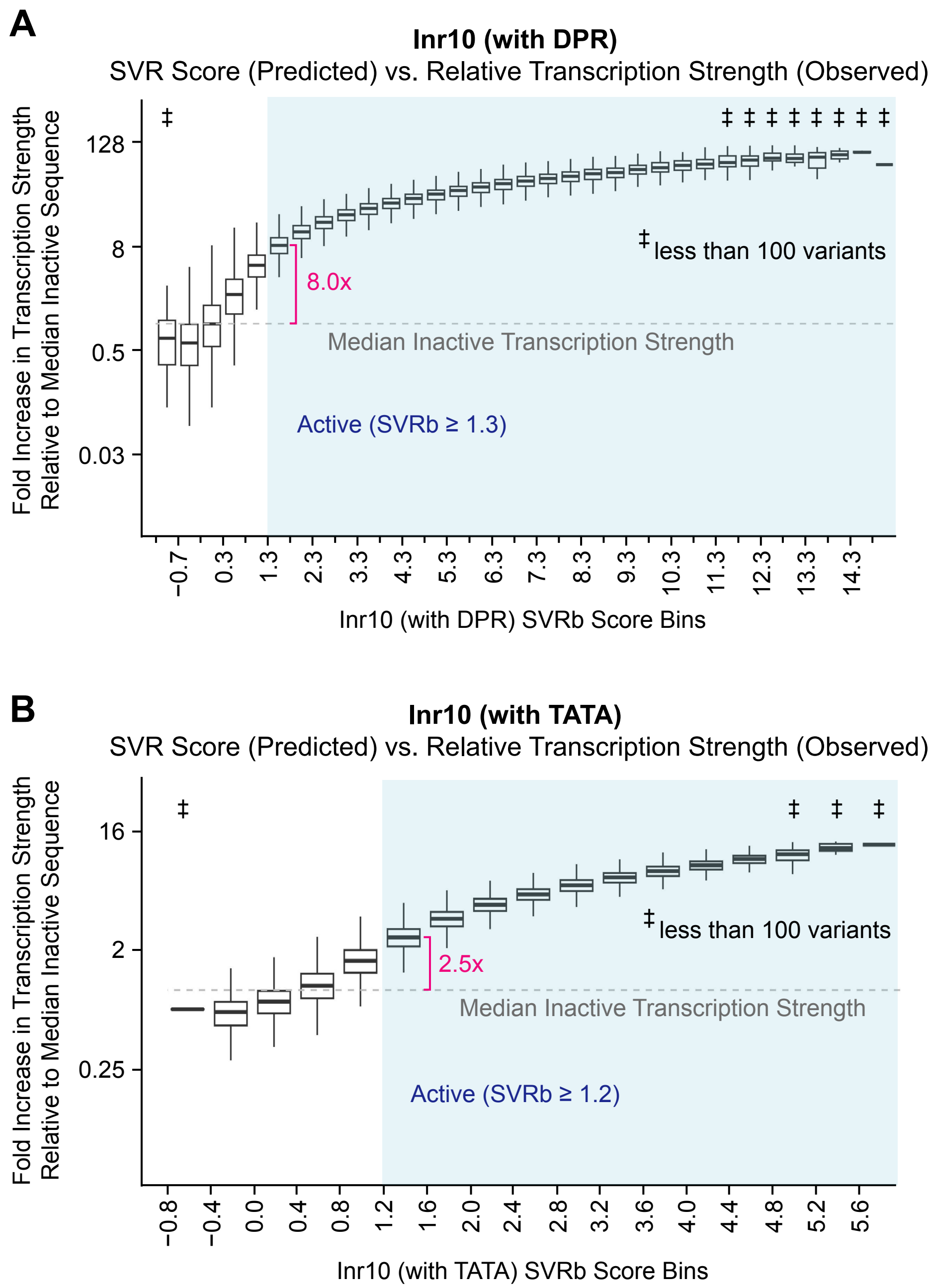

Supplemental Figure S19. Inr sequence variants exhibit a broader range of transcriptional activity in DPR-driven promoters than in TATA-driven promoters. Shown are box-plot diagrams of the transcription strengths of all HARPE sequence variants placed into bins of the indicated SVR score ranges. (*A*) Sequence variants with Inr10 (with DPR) SVRb scores ≥ 1.3 are typically at least 8 times more active than an inactive sequence. (*B*) Sequence variants with Inr10 (with TATA) SVRb scores ≥ 1.2 are typically at least 2.5 times more active than an inactive sequence. In both diagrams, the thick horizontal lines are the medians, and the lower and upper hinges are the first and third quartiles, respectively. Whiskers extend from the hinges to the largest or lowest value no further than 1.5 * IQR from the hinge. Data beyond the end of the whiskers (outlying points) are omitted from the box-plots. Variants with transcription strength = 0 were removed to allow log-scale display of the diagram. The light blue shaded regions indicate sequence variants with SVR scores greater than or equal to the optimal SVR thresholds identified in Supplemental Fig. S7. The horizontal dashed grey line denotes the median transcription strength of inactive sequences.

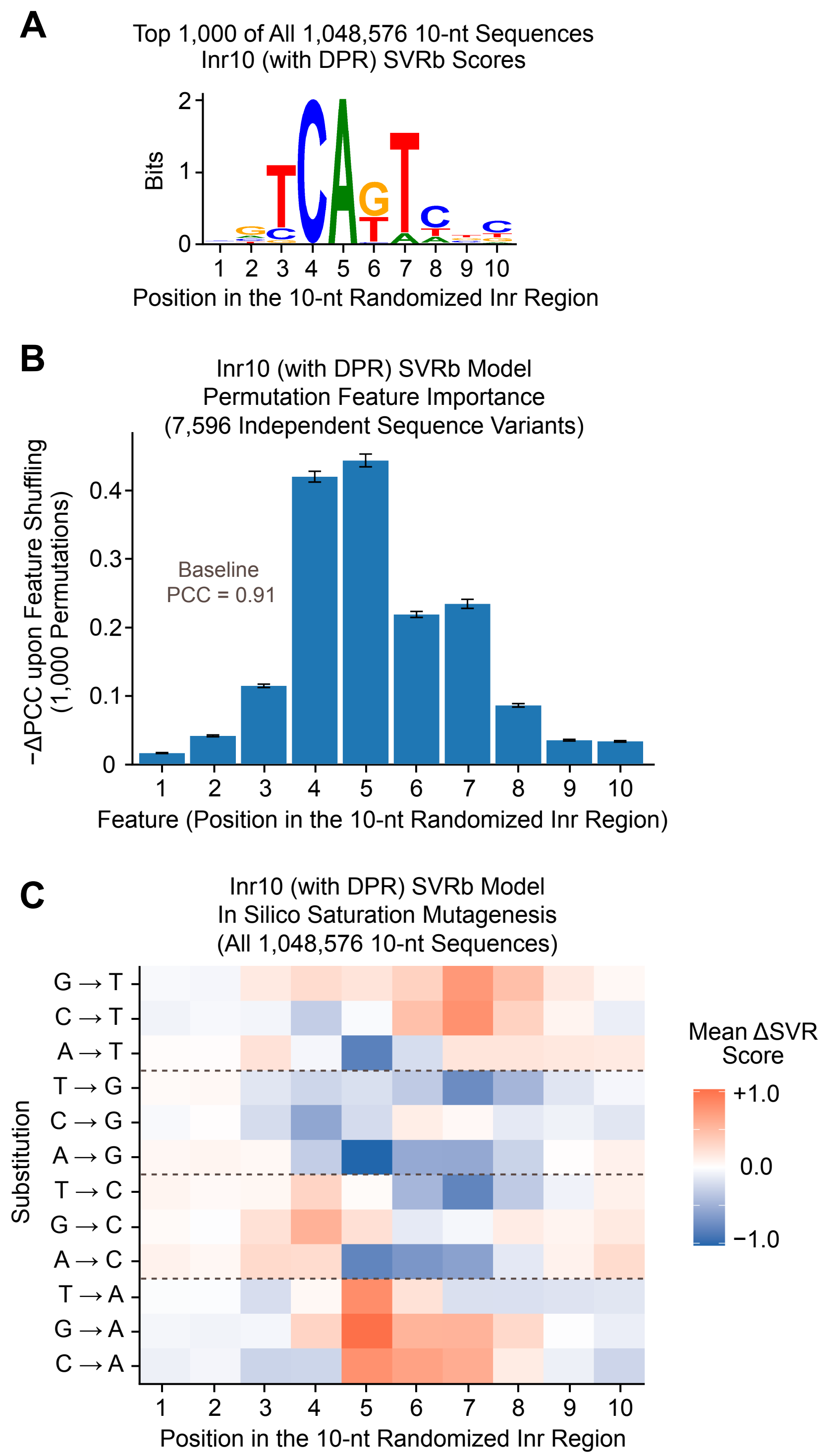

Supplemental Figure S20. Model interpretation analyses reveal key sequence features learned by the Inr10 (with DPR) SVRb model. (*A*) SVR analysis of all possible 10-nt sequences reveals the predicted optimal Inr motif. Shown is the web logo of the predicted top 1,000 out of all 1,048,576 10-nt sequences. (*B*) Permutation feature importance analysis reveals that positions 4 to 7, which correspond to the core Inr CAKT motif, are most important for SVR prediction of transcriptional activity. Each feature (position in the 10-nt randomized Inr region) was shuffled between the 7,596 independent test sequences shown in Fig. 1D (left), and the resulting decrease in model performance [as indicated by the change in Pearson's correlation coefficient (PCC) between the observed transcription strengths and predicted SVR scores] was measured. The baseline model performance (PCC = 0.91) is shown in Fig. 1D (left). (*C*) In silico saturation mutagenesis reveals position- and nucleotide-specific effects on predicted transcriptional activity. Each position in the 10-nt randomized Inr region was systematically mutated across all possible sequences to each of the three alternative nucleotides. Shown is the mean change in SVR score for each substitution.

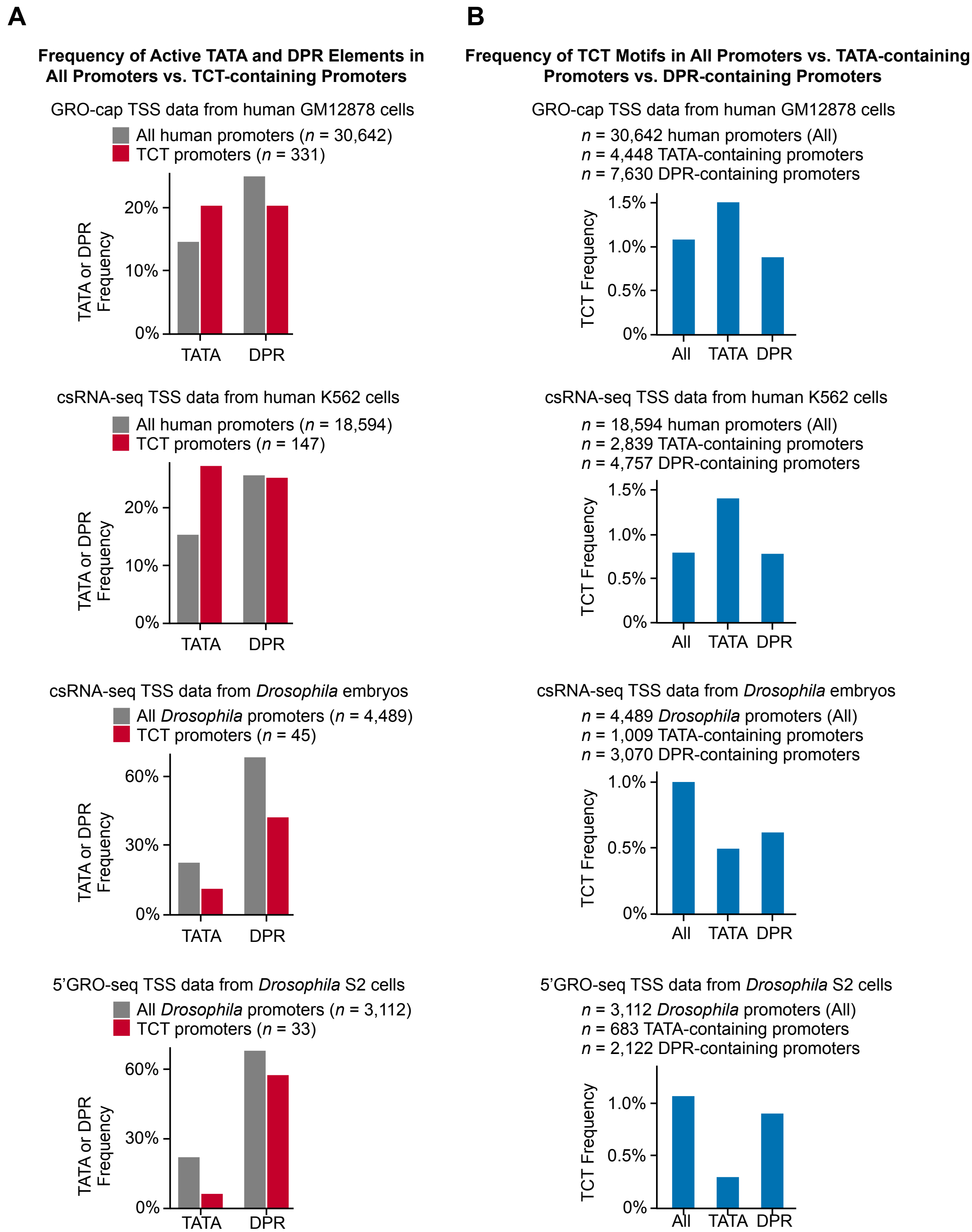

Supplemental Figure S21. The TCT motif functions differently in humans than in *Drosophila*. (*A*) The TATA box is overrepresented in human TCT promoters and is underrrepresented in *Drosophila* TCT promoters. The predicted frequencies of active TATA and DPR elements in all identified promoters versus only TCT-containing promoters are shown. (*B*) The TCT motif is enriched in human TATA-containing promoters and is depleted in *Drosophila* TATA-containing promoters. The frequencies of TCT motifs in all identified promoters (All), TATA-containing promoters, and DPR-containing promoters are shown. The predicted activities of the TATA and DPR elements were determined by using SVR models of the human TATA box (Vo ngoc et al. 2020) and the human and *Drosophila* DPR (Vo ngoc et al. 2020, 2023). TCT motifs were identified by a match to the TCT consensus sequence (YC_{+1}TYTYY in humans and YYC_{+1}TTTYY in *Drosophila*).

Supplemental Table 1

Promoter Construct	HARPE Experiment Randomized Region	Biochemical or Cells	Reproducibility (PCC) (transcription strength; rep1 vs rep 2)
Inr10 (with DPR)	10-nt	Biochemical	0.94
Inr10 (with TATA)	10-nt	Biochemical	0.91
Inr14 (with DPR)	14-nt	Biochemical	0.96
Inr14 (with TATA)	14-nt	Biochemical	0.89
Inr14 (Inr only)	14-nt	Biochemical	0.71, 0.60, 0.62 *
Inr14 (with DPR)	14-nt	Cells	0.55
Inr14 (with TATA)	14-nt	Cells	0.63

*Due to low transcription levels with this promoter, we performed 3 replicates.
The three PCC values are for rep1 vs. rep2, rep2 vs. rep3, and rep1 vs. rep3.

Supplemental Table 2

GM12878 cells (GRO-cap TSS data)						
**Human Promoter Types**	**% Active Inr10 (with DPR)**	**% Active Inr10 (with TATA)**	**% Active Inr (either)***	**% Active Inr (both)****	**% Active TATA**	**% Active DPR**
All	62.2	65.2	70	57.3	14.5	24.9
Active Inr10 (with DPR)	[100]	92.1	[100]	92.1	12.7	25.9
Active Inr10 (with TATA)	87.9	[100]	[100]	87.9	13.7	25.4
Active Inr (either)*	88.8	93	[100]	81.8	13.5	25.4
Active Inr (both)**	[100]	[100]	[100]	[100]	12.7	25.9
Inactive Inr (both)***	[0]	[0]	[0]	[0]	16.8	23.7
Active TATA	54.1	61.3	65.3	50.1	[100]	20.8
Inactive TATA	63.5	65.8	70.8	58.5	[0]	25.6
Active DPR	64.6	66.4	71.5	59.5	12.1	[100]
Inactive DPR	61.4	64.7	69.5	56.6	15.3	[0]

*Active with the Inr10 (with TATA) SVR model and/or the Inr10 (with DPR) SVR model
**Active with both the Inr10 (with TATA) SVR model and the Inr10 (with DPR) SVR model
***Inactive with both the Inr10 (with TATA) SVR model and the Inr10 (with DPR) SVR model

K562 cells (csRNA-seq TSS data)						
**Human Promoter Types**	**% Active Inr10 (with DPR)**	**% Active Inr10 (with TATA)**	**% Active Inr (either)***	**% Active Inr (both)****	**% Active TATA**	**% Active DPR**
All	58.4	58.1	64.3	52.2	15.3	25.6
Active Inr10 (with DPR)	[100]	89.4	[100]	89.4	13.4	27.1
Active Inr10 (with TATA)	89.8	[100]	[100]	89.8	13.9	26.7
Active Inr (either)*	90.8	90.4	[100]	81.1	14	26.7
Active Inr (both)**	[100]	[100]	[100]	[100]	13.2	27.2
Inactive Inr (both)***	[0]	[0]	[0]	[0]	17.5	23.6
Active TATA	51.2	53.0	59.0	45.1	[100]	21.3
Inactive TATA	59.7	59.1	65.3	53.5	[0]	26.4
Active DPR	61.8	60.7	67.1	55.5	12.7	[100]
Inactive DPR	57.2	57.3	63.4	51.1	16.2	[0]

*Active with the Inr10 (with TATA) SVR model and/or the Inr10 (with DPR) SVR model
**Active with both the Inr10 (with TATA) SVR model and the Inr10 (with DPR) SVR model
***Inactive with both the Inr10 (with TATA) SVR model and the Inr10 (with DPR) SVR model

Drosophila embryos (csRNA-seq TSS data)****						
***Drosophila* Promoter Types**	**% Active Inr10 (with DPR)**	**% Active Inr10 (with TATA)**	**% Active Inr (either)***	**% Active Inr (both)****	**% Active TATA**	**% Active DPR**
All	86.6	87.9	90.2	84.3	22.5	68.4
Active Inr10 (with DPR)	[100]	97.2	[100]	97.2	21.3	75.9
Active Inr10 (with TATA)	95.9	[100]	[100]	95.9	22	74.1
Active Inr (either)*	96	97.4	[100]	93.4	22	73.6
Active Inr (both)**	[100]	[100]	[100]	[100]	21.3	76.6
Inactive Inr (both)***	[0]	[0]	[0]	[0]	26.7	20.5
Active TATA	82.3	86	88.4	79.9	[100]	61.3
Inactive TATA	87.9	88.4	90.8	85.5	[0]	70.4
Active DPR	96.2	95.2	97.1	94.3	20.2	[100]
Inactive DPR	66.0	72.0	75.5	62.4	27.5	[0]

*Active with the human Inr10 (with TATA) SVR model and/or the human Inr10 (with DPR) SVR model
**Active with both the human Inr10 (with TATA) SVR model and the human Inr10 (with DPR) SVR model
***Inactive with both the Inr10 (with TATA) SVR model and the Inr10 (with DPR) SVR model
****Using the *Drosophila* DPR SVR model and the human Inr10 & TATA SVR models

S2 cells (5'GRO-seq TSS data)****						
***Drosophila* Promoter Types**	**% Active Inr10 (with DPR)**	**% Active Inr10 (with TATA)**	**% Active Inr (either)***	**% Active Inr (both)****	**% Active TATA**	**% Active DPR**
All	86.7	87.2	89.1	84.8	21.9	68.2
Active Inr10 (with DPR)	[100]	97.8	[100]	97.8	20.9	74.6
Active Inr10 (with TATA)	97.9	[100]	[100]	97.9	21.1	74.0
Active Inr (either)*	97.3	97.8	[100]	95.1	21.3	73.2
Active Inr (both)**	[100]	[100]	[100]	[100]	20.8	74.8
Inactive Inr (both)***	[0]	[0]	[0]	[0]	27.5	26.9
Active TATA	82.6	84.3	86.4	80.5	[100]	63.1
Inactive TATA	87.9	88.0	89.9	86.0	[0]	69.6
Active DPR	94.8	93.9	95.7	93.0	20.3	[100]
Inactive DPR	69.3	72.9	75.1	67.2	25.5	[0]

*Active with the human Inr10 (with TATA) SVR model and/or the human Inr10 (with DPR) SVR model
**Active with both the human Inr10 (with TATA) SVR model and the human Inr10 (with DPR) SVR model
***Inactive with both the Inr10 (with TATA) SVR model and the Inr10 (with DPR) SVR model
****Using the *Drosophila* DPR SVR model and the human Inr10 & TATA SVR models

Supplemental Table 3A

**TSS Dataset:**	**K562 Cells (csRNA-seq; Duttke et al. 2019)**
**Annotation:**	**GENCODE (v49)**
**Promoters:**	**Contain Perfect Match to the TCT Motif (YC+1TYTYY)**
	**Ranked by Inr14 (with DPR) SVRc score**

Sequence from -5 to +9	Inr14 (with DPR) SVRc Score	Gene.Name
CCTTCCTTTTCCTC	4.73	RPLP2
GCTTCCTTTTCCGG	4.58	SYNJ2BP
GCTTCCTTTTTGTC	4.27	RPS25
CTTTCCTTTTCCGG	4.24	RPS16
GCTTCCTTTCTCTC	4.21	RPS21
CCTTCCTTTTTCGT	4.19	RPL27A
ACTTCCTTTTCCCC	4.11	SEH1L
GTTTCCTTTTTCCT	4.06	SSBP1
GCTTCCTTTTTCCG	4.04	ENSG00000287620
GCTTCCTTTTCGAT	4.04	RPS27AP12
ACGTTCTTTTTCGC	3.94	EEF1A1
GTTTCCTTTCCGTG	3.88	RPSAP3
TCTTCCTTTCCCGC	3.86	SNHG6
CCTCCCTTTTCGGC	3.84	SNHG26
TCGCCCTTTTCGGC	3.83	CDK7
TCGGCCTTTTCCGC	3.77	TPT1
CTCTCCTTTTCGTT	3.71	CFL1
ACTTCCTTTCTCGG	3.7	TAOK3
GTTTCCTTTCCCCG	3.7	MYL5
CCGCTCTTTTCCGG	3.57	RPL10AP6
TTTCTCTTTCCGCG	3.52	SOCS1
CAGGCCTTTCCGCT	3.51	RPL13P12
AGCTTCTTTTCGGC	3.5	RPL21P28
TCTCTCTTTCCGGA	3.45	RPL18
TCTCTCTTTCTGGC	3.4	B2M
TGCCTCTTTCTGAG	3.39	SNAPC2
TCCTCCTTTCCCTG	3.38	TEX19
ACTCTCTTTTCCGG	3.33	RPL7P16
CACGCCTTTTCCGG	3.31	POLR1E
GCGTTCTTTTCGAG	3.29	SNHG16
GCCCTCTTTCCCTG	3.27	TRIM52-AS1
CCGCCCTTTCTGTG	3.17	SREBF2
CCTTTCTTTTTGAG	3.14	RPS20
CTGCTCTTTTCGAT	3.09	HNRNPL
TAGTCCTTTCTCGT	3.06	TMED2-DT
TCTTCCTTTTCAGT	3.04	CHEK2
GGGTCCTTTTTCCT	3	EEF1B2
GCCGCCTTTCTCCG	2.95	CNIH1
GAGCTCTTTTCTGA	2.94	RPS15P5
CCGGTCTTTCTGGT	2.94	RPL37
ACCCTCTTTCTGGC	2.89	ENSG00000290812
CCTTTCTTTTTTTC	2.86	RPS11
CTCTCCTTTTTGGA	2.77	RCC1
ACTGTCTTTTCGAG	2.77	GAS5
CCTGTCTTTTCGGG	2.77	SNHG19
AACGCCTTTTCGGG	2.76	MRPL54
CCTTTCTCTTCCGG	2.74	RPL29
GCTCTCTCTTCCAC	2.74	RPS18
ACTCCCTTTTCTTT	2.74	EIF3E
CCCGCCTTTTCTCT	2.73	CIAO2A
TGTTCCTTTCCCCT	2.73	MAN2A1
TCAGCCTTTCTGTG	2.72	ANXA2R-OT1
AGGTTCTTTCTGTG	2.71	MRPS16
CACCTCTCTTCCGG	2.7	LINC02323
CCCGCCTTTTTTGC	2.7	CHCHD10
GCGTTCTCTTCCGC	2.66	EEF2
GCCCTCTCTTCCTG	2.64	RHOC
CCGCCCTTTCCTGT	2.64	KXD1
GCGGTCTTTCTCTA	2.63	SNX5
ACCGTCTTTCCTGG	2.61	PTPRH
TAAGTCTTTCTGGA	2.61	GCFC2
TGCTTCTTTCCCAA	2.57	RPL13AP20
CCTTCCTCTTTCCG	2.56	RPL28
CCGCTCTCTTCCGG	2.55	FAAP100
TACCCCTTTTTCCC	2.55	PWWP3A
GCGGCCTTTCCCAC	2.53	DLEU2
CAACCCTTTTCTTC	2.51	TAF1D
GAGGCCTTTCCGGA	2.5	RCCD1
TCGTTCTCTTCGCG	2.49	SNX3
CCCTTCTCTTTCGG	2.48	ENSG00000269176
TCAGCCTTTTCCAG	2.47	ZBTB48
GCGGCCTTTTTTTC	2.43	CERS2
CAGGCCTTTCCTTA	2.41	KRT8
TCTTCCTCTTTCCC	2.39	RPL17
TGTCTCTCTTCGGG	2.35	NOP14
CGCGTCTCTTTCGC	2.32	SND1
CCGATCTTTCTGAA	2.31	ENSG00000295453
TGAGCCTTTCCTTT	2.26	ENSG00000218227
TTGGTCTTTCCTGC	2.22	LINC02780
TGGGCCTTTTCTCT	2.2	DPH5
ACTCTCTCTCTCTC	2.19	LINC00958
CCGTCCTCTTTCCG	2.18	RPS15A
CGGCTCTCTCTGCG	2.18	PIP5K1B
TCTTCCTCTTCCAG	2.15	ENSG00000231416
TCGGCCTCTTTGCG	2.13	RPA2
GCTACCTTTTCCCA	2.13	ZRANB3
CCGCCCTCTTCGTC	2.13	HCG18
CGCTTCTCTTTCTG	2.09	UQCRB
TTCATCTTTCCGGA	2.05	ENSG00000240311
CTTCTCTCTTTTCC	2.04	ENSG00000227681
TTGCTCTCTTCCGG	2.02	COX4I1
TCTTTCTCTTTGGG	1.99	ID3
CGCCTCTCTCCGCC	1.97	PABPC4
GCTTCCTCTCTGCT	1.91	RPL22L1
GCGGCCTCTTCCTG	1.88	RPS5
ACCCCCTTTCTTTG	1.87	EEF1G
CCTCTCTCTTTACT	1.86	CANX
AAACTCTCTTCTGG	1.86	BPGM
CGCGCCTCTTTCTC	1.85	RPS9
TTTTTCTTTCCAAA	1.85	LYRM4
CGCTTCTCTCTGGC	1.76	STK16
CCGCCCTCTTTCCC	1.76	TRIM52-AS1
TCTTCCTCTTTTCT	1.76	GBGT1
TCTCCCTCTCTGAA	1.7	SP3
TTAACCTTTCCGGC	1.69	RPS27P19
GGCTCCTCTTTGAT	1.67	C5orf34-AS1
TCGGTCTCTTTTCG	1.66	CERS2
CGGATCTCTTCGTC	1.61	PKM
GTGACCTTTTCATT	1.61	SNHG29
GCGATCTCTTCTGA	1.61	RPS15
AAGCTCTCTTTTTG	1.58	HBE1
TAACTCTCTTTTCC	1.58	RPL7P16
GTTGCCTCTTTTTC	1.55	RPL7P24
TGGGTCTCTTCCTA	1.51	RPS7
CTAGTCTCTTTTCC	1.47	RPL10A
TGGTTCTCTTTTCC	1.46	RPS24
CTGGTCTCTCTGCA	1.41	ERMAP
CGCGCCTCTCTGTC	1.29	PPDPF
CCAGCCTCTTTTTC	1.26	RPL7P50
TGGCCCTCTTTTCC	1.14	RPS6
GCTGTCTCTTTAAG	1.09	ITPRIPL1
CTACCCTCTTCTGT	1.01	LCMT1
GCACCCTCTCCCCT	0.48	SUMO2

ribosomal protein genes
translation factors

Supplemental Table 3B

**TSS Dataset:**	**K562 Cells (csRNA-seq; Duttke et al. 2019)**
**Annotation:**	**GENCODE (v49)**
**Promoters:**	**Contain Perfect Match to the TCT Motif (YC+1TYTYY)**
	**Ranked by Inr14 (with TATA) SVRc score**

Sequence from -5 to +9	Inr14 (with TATA) SVRc Score	Gene.Name
GCTTCCTTTTCCGG	2.61	SYNJ2BP
CCTTCCTTTTCCTC	2.5	RPLP2
TCTTCCTTTCCCGC	2.47	SNHG6
ACTTCCTTTTCCCC	2.45	SEH1L
TCGGCCTTTTCCGC	2.44	TPT1
TCTCTCTTTCCGGA	2.44	RPL18
GCTTCCTTTTTCCG	2.43	ENSG00000287620
GCTTCCTTTCTCTC	2.42	RPS21
GCTTCCTTTTCGAT	2.33	RPS27AP12
ACTCTCTTTTCCGG	2.32	RPL7P16
CTTTCCTTTTCCGG	2.3	RPS16
TCTTCCTCTTTCCC	2.29	RPL17
GTTTCCTTTTTCCT	2.26	SSBP1
CCTTCCTTTTTCGT	2.25	RPL27A
GCTTCCTTTTTGTC	2.22	RPS25
ACGTTCTTTTTCGC	2.19	EEF1A1
TCTCTCTTTCTGGC	2.18	B2M
GCGTTCTTTTCGAG	2.18	SNHG16
GAGCTCTTTTCTGA	2.17	RPS15P5
CCTCCCTTTTCGGC	2.16	SNHG26
ACTTCCTTTCTCGG	2.15	TAOK3
TCAGCCTTTTCCAG	2.14	ZBTB48
AGCTTCTTTTCGGC	2.14	RPL21P28
CCTTTCTTTTTGAG	2.14	RPS20
CACGCCTTTTCCGG	2.14	POLR1E
CCGCTCTTTTCCGG	2.12	RPL10AP6
CTTCTCTCTTTTCC	2.1	ENSG00000227681
CCTGTCTTTTCGGG	2.09	SNHG19
TTGCTCTCTTCCGG	2.08	COX4I1
GTTTCCTTTCCCCG	2.07	MYL5
TCTTTCTCTTTGGG	2.06	ID3
TTTCTCTTTCCGCG	2.06	SOCS1
TCGCCCTTTTCGGC	2.06	CDK7
CCCGCCTTTTTTGC	2.05	CHCHD10
ACTCTCTCTCTCTC	2.03	LINC00958
CAACCCTTTTCTTC	2.03	TAF1D
CAGGCCTTTCCTTA	2.03	KRT8
CCTTCCTCTTTCCG	2.03	RPL28
TTGGTCTTTCCTGC	2.02	LINC02780
TAAGTCTTTCTGGA	2.02	GCFC2
CCGCCCTTTCCTGT	2.01	KXD1
TCTTCCTTTTCAGT	2	CHEK2
GCTCTCTCTTCCAC	2	RPS18
TCTTCCTCTTCCAG	1.99	ENSG00000231416
ACTGTCTTTTCGAG	1.99	GAS5
CTGCTCTTTTCGAT	1.98	HNRNPL
GGGTCCTTTTTCCT	1.98	EEF1B2
CCTTTCTCTTCCGG	1.98	RPL29
CTCTCCTTTTCGTT	1.97	CFL1
GCCGCCTTTCTCCG	1.97	CNIH1
GCGTTCTCTTCCGC	1.97	EEF2
TAGTCCTTTCTCGT	1.95	TMED2-DT
GTTTCCTTTCCGTG	1.95	RPSAP3
GCCCTCTCTTCCTG	1.94	RHOC
CTAGTCTCTTTTCC	1.94	RPL10A
CCTTTCTTTTTTTC	1.93	RPS11
GCGGCCTTTCCCAC	1.92	DLEU2
CGCGTCTCTTTCGC	1.92	SND1
CGCTTCTCTTTCTG	1.92	UQCRB
CGCGCCTCTTTCTC	1.91	RPS9
TAACTCTCTTTTCC	1.91	RPL7P16
TCAGCCTTTCTGTG	1.91	ANXA2R-OT1
CCGCCCTCTTTCCC	1.91	TRIM52-AS1
TCGTTCTCTTCGCG	1.91	SNX3
ACTCCCTTTTCTTT	1.9	EIF3E
TGTCTCTCTTCGGG	1.89	NOP14
TGTTCCTTTCCCCT	1.89	MAN2A1
TGCTTCTTTCCCAA	1.88	RPL13AP20
AACGCCTTTTCGGG	1.88	MRPL54
GCTACCTTTTCCCA	1.88	ZRANB3
CCGGTCTTTCTGGT	1.87	RPL37
TTTTTCTTTCCAAA	1.87	LYRM4
CCCTTCTCTTTCGG	1.86	ENSG00000269176
GAGGCCTTTCCGGA	1.85	RCCD1
CCGTCCTCTTTCCG	1.83	RPS15A
ACCGTCTTTCCTGG	1.83	PTPRH
TGAGCCTTTCCTTT	1.83	ENSG00000218227
AGGTTCTTTCTGTG	1.82	MRPS16
CCTCTCTCTTTACT	1.82	CANX
CCGCTCTCTTCCGG	1.81	FAAP100
TCCTCCTTTCCCTG	1.81	TEX19
CAGGCCTTTCCGCT	1.79	RPL13P12
GCGATCTCTTCTGA	1.79	RPS15
TGCCTCTTTCTGAG	1.79	SNAPC2
TCGGCCTCTTTGCG	1.77	RPA2
GCGGTCTTTCTCTA	1.77	SNX5
CTCTCCTTTTTGGA	1.74	RCC1
TCTCCCTCTCTGAA	1.74	SP3
TCGGTCTCTTTTCG	1.73	CERS2
GCGGCCTCTTCCTG	1.73	RPS5
GCCCTCTTTCCCTG	1.73	TRIM52-AS1
TACCCCTTTTTCCC	1.71	PWWP3A
CCAGCCTCTTTTTC	1.71	RPL7P50
CCCGCCTTTTCTCT	1.7	CIAO2A
GCTTCCTCTCTGCT	1.68	RPL22L1
CCGCCCTCTTCGTC	1.68	HCG18
TTCATCTTTCCGGA	1.67	ENSG00000240311
CCGCCCTTTCTGTG	1.67	SREBF2
AAACTCTCTTCTGG	1.66	BPGM
TCTTCCTCTTTTCT	1.66	GBGT1
CGGCTCTCTCTGCG	1.65	PIP5K1B
AAGCTCTCTTTTTG	1.63	HBE1
TGGTTCTCTTTTCC	1.62	RPS24
CACCTCTCTTCCGG	1.61	LINC02323
GGCTCCTCTTTGAT	1.61	C5orf34-AS1
GCGGCCTTTTTTTC	1.57	CERS2
CGGATCTCTTCGTC	1.55	PKM
GCTGTCTCTTTAAG	1.55	ITPRIPL1
TGGCCCTCTTTTCC	1.55	RPS6
TGGGCCTTTTCTCT	1.54	DPH5
TGGGTCTCTTCCTA	1.54	RPS7
TTAACCTTTCCGGC	1.53	RPS27P19
CTGGTCTCTCTGCA	1.51	ERMAP
CCGATCTTTCTGAA	1.51	ENSG00000295453
ACCCTCTTTCTGGC	1.48	ENSG00000290812
GTTGCCTCTTTTTC	1.45	RPL7P24
GTGACCTTTTCATT	1.42	SNHG29
CTACCCTCTTCTGT	1.39	LCMT1
CGCTTCTCTCTGGC	1.35	STK16
CGCCTCTCTCCGCC	1.34	PABPC4
CGCGCCTCTCTGTC	1.33	PPDPF
ACCCCCTTTCTTTG	1.31	EEF1G
GCACCCTCTCCCCT	1.16	SUMO2

ribosomal protein genes (green highlight)
translation factors (blue highlight)

Supplemental Table 4A

TSS dataset:	GM12878 (GRO-cap; Core et al. 2014)
Annotation:	GENCODE (v19)
Promoters:	TATA only (TATA+, Inr-, DPR-)
**GO analysis:**	**ORA (Over-Representation Analysis), top 25 hits**
The GO terms are ranked by the p.adjust values.	

ID	Description	GeneRatio	BgRatio	p.adjust
GO:0010628	positive regulation of gene expression	68/498	491/6988	0.00014
GO:0034097	response to cytokine	58/498	406/6988	0.00027
GO:0071345	cellular response to cytokine stimulus	48/498	361/6988	0.01707
GO:0006338	chromatin remodeling	47/498	358/6988	0.02115
GO:0070848	response to growth factor	35/498	240/6988	0.02115
GO:0000165	MAPK cascade	40/498	291/6988	0.02115
GO:0030857	negative regulation of epithelial cell differentiation	6/498	11/6988	0.02132
GO:0043408	regulation of MAPK cascade	34/498	237/6988	0.02473
GO:0071363	cellular response to growth factor stimulus	33/498	228/6988	0.02473
GO:0001656	metanephros development	8/498	22/6988	0.02721
GO:0048871	multicellular organismal-level homeostasis	43/498	334/6988	0.02721
GO:0043405	regulation of MAP kinase activity	13/498	55/6988	0.02721
GO:0060993	kidney morphogenesis	8/498	23/6988	0.02969
GO:0006979	response to oxidative stress	29/498	196/6988	0.02969
GO:0072028	nephron morphogenesis	7/498	18/6988	0.03083
GO:0072088	nephron epithelium morphogenesis	7/498	18/6988	0.03083
GO:0043410	positive regulation of MAPK cascade	24/498	155/6988	0.04242
GO:0045648	positive regulation of erythrocyte differentiation	6/498	14/6988	0.04242
GO:0097190	apoptotic signaling pathway	40/498	317/6988	0.04242
GO:0034612	response to tumor necrosis factor	19/498	111/6988	0.04242
GO:0038034	signal transduction in absence of ligand	9/498	32/6988	0.04242
GO:0097192	extrinsic apoptotic signaling pathway in absence of ligand	9/498	32/6988	0.04242
GO:0006334	nucleosome assembly	11/498	46/6988	0.04242
GO:0000302	response to reactive oxygen species	17/498	94/6988	0.04242
GO:0009612	response to mechanical stimulus	14/498	70/6988	0.04641

GeneRatio	fraction of input genes that are annotated to each GO term
BgRatio	fraction of background ("universe") genes that are annotated to each GO term
p.adjust	significance after Benjamini–Hochberg correction for multiple comparisons

Supplemental Table 4B

TSS dataset:	GM12878 (GRO-cap; Core et al. 2014)
Annotation:	GENCODE (v19)
Promoters:	Ranked by TATA SVR score
**GO analysis:**	**GSEA (Gene Set Enrichment Analysis), top 25 hits**

The GO terms are ranked by the p.adjust values.

ID	Description	setSize	NES	p.adjust
GO:0006334	nucleosome assembly	46/6988	1.660116244	0.00008
GO:0034728	nucleosome organization	60/6988	1.621213769	0.00008
GO:0006338	chromatin remodeling	358/6988	1.328898765	0.00008
GO:0065004	protein-DNA complex assembly	121/6988	1.465126199	0.00018
GO:0048871	multicellular organismal-level homeostasis	334/6988	1.313360986	0.00051
GO:1903706	regulation of hemopoiesis	197/6988	1.374269269	0.00058
GO:0060284	regulation of cell development	333/6988	1.31177516	0.00058
GO:0006325	chromatin organization	443/6988	1.276592398	0.00058
GO:0000122	negative regulation of transcription by RNA polymerase II	415/6988	1.275706262	0.00058
GO:0071363	cellular response to growth factor stimulus	228/6988	1.350200719	0.00072
GO:0071345	cellular response to cytokine stimulus	361/6988	1.291639409	0.00084
GO:0060429	epithelium development	382/6988	1.282729569	0.00084
GO:0034097	response to cytokine	406/6988	1.278057771	0.00084
GO:0009628	response to abiotic stimulus	474/6988	1.263211652	0.00084
GO:0007155	cell adhesion	479/6988	1.256588566	0.00089
GO:0010628	positive regulation of gene expression	491/6988	1.254221993	0.00117
GO:0045910	negative regulation of DNA recombination	32/6988	1.648419289	0.00118
GO:0040011	locomotion	405/6988	1.273776095	0.00123
GO:0070848	response to growth factor	240/6988	1.332514242	0.00124
GO:0001944	vasculature development	244/6988	1.314518151	0.00158
GO:0051093	negative regulation of developmental process	323/6988	1.287451761	0.00158
GO:0030261	chromosome condensation	28/6988	1.660573869	0.00162
GO:1902105	regulation of leukocyte differentiation	158/6988	1.379658267	0.00162
GO:0048514	blood vessel morphogenesis	198/6988	1.333561384	0.00162
GO:0008285	negative regulation of cell population proliferation	267/6988	1.308574753	0.00162

setSize	fraction of genes from the ranked list that are annotated to each GO term
NES	(normalized enrichment score) measure of the strength/direction of a GO term's distribution in the ranked list, with positive values indicating clustering towards the top
p.adjust	significance after Benjamini–Hochberg correction for multiple comparisons

Supplemental Materials and Methods

HARPE library generation

The Inr HARPE libraries were generated as described in Vo ngoc et al. (2020), except that some libraries were prepared by electroporation with MegaX DH10B T1R Electrocomp Cells (Thermo Fisher Scientific) instead of DH5G CloneCatcher Gold Electrocompetent Cells (Genlantis). The HARPE vector and SCP1-based promoter backbones, which include constructs with mutations in the DPR and/or the TATA box, are identical to those used previously (Vo ngoc et al. 2020, 2023). For mutation of the DPR, the sequence AGACGTG from +28 to +34 relative to the SCP1 +1 TSS was mutated to CTCATGT. For mutation of the TATA box, the sequence TATATAAG from −31 to −24 was mutated to ACGTCCGT. As a reference, these mutations decreased the human DPR and TATA SVR scores from 14.59 to 0.85 and from 16.18 to 0.09, respectively. For HARPE analysis of the Inr, the 14-nt region from −5 to +9 and the 10-nt region from −4 to +6 (relative to the SCP1 +1 TSS) were randomized to generate ~500,000 Inr sequence variants within SCP1 promoter backbones containing a DPR (mutTATA), a TATA box (mutDPR), or neither element (mutTATA, mutDPR). With the 10-nt libraries, it was necessary to generate ~1,000,000 independent transformants to achieve ~500,000 different Inr sequence variants because there are only 1,048,576 (= 4^{10}) possible sequences in a randomized 10-mer, and we obtained many duplicate Inr sequences. The HARPE transcription reactions were scaled according to the number of independent transformants. To enable the use of HARPE for the Inr region, which includes sequence that are both upstream and downstream of the transcription start site, a downstream barcode was added by randomizing the region from +53 to +69 or +53 to +67 (relative to the SCP1 +1 TSS) for the 14-nt or 10-nt randomized Inr libraries, respectively, as was done for the HARPE analysis of the TATA box (Vo ngoc et al. 2020).

Transcription of HARPE libraries

In the biochemical HARPE experiments, the plasmid libraries were subjected to in vitro transcription with HeLa nuclear extracts (6 or 12 standard reactions for the 14-nt or 10-nt randomized Inr libraries, respectively; as noted above, the reactions were scaled according to the number of independent transformants in each library), as described in Vo ngoc et al. (2020). For each reaction, we used 8 μL of nuclear extract (7 mg protein/mL), which was prepared by the method of Dignam et al. (1983). The RNA from the 6 or 12 reactions was combined and processed as described below. In the cell-based HARPE experiments, the plasmid libraries were transfected into HeLa cells [five 10-cm culture dishes per library (14-nt randomized Inr libraries only)] as in Vo ngoc et al. (2020). During collection, one-fifth of each cell pellet was reserved for plasmid DNA extraction, and the remainder of the cells was used for RNA extraction. All HARPE experiments were performed independently at least two times to ensure reproducibility of the data. Replicates were performed with the same HARPE DNA libraries in independent transcription reactions or transfection analyses.

HARPE RNA extraction and processing

RNA transcripts from cells or from in vitro transcription reactions were extracted with Trizol or Trizol LS (Thermo Fisher Scientific), respectively. Total RNA (120 μg per condition for cell transfection experiments, or the entirety of each condition for in vitro transcription experiments) was processed as described in Vo ngoc et al. (2020, 2023). Briefly, the following steps were performed. First, contaminating plasmid DNA was removed with the TURBO DNA-free Kit (Thermo Fisher Scientific). Next, the RNA was subjected to reverse transcription with SuperScript III Reverse Transcriptase (Thermo Fisher Scientific) and treatment with RNase H (New England Biolabs). The resulting cDNA was size-selected on a 6% (w/v) polyacrylamide-8 M urea gel with fluorescently-labeled size markers. The size-selected cDNAs and HARPE plasmid DNAs were used as templates to generate DNA amplicons for Illumina sequencing (RNA and DNA datasets, respectively). In the cell-based HARPE experiments, the DNA

datasets were obtained with plasmid DNA samples that were extracted post-transfection. Custom forward oligonucleotides containing the Illumina P5 and Read1-primer sequences preceding the sequences corresponding to nucleotides +19 to +34 (relative to the SCP1 +1 TSS; the SCP1 DPR and mutDPR promoter backbones required different primers) were used to amplify the randomized barcode regions. Custom forward oligonucleotides containing the Illumina P5 and Read1-primer sequences preceding the sequences corresponding to nucleotides −44 to −23 or −40 to −23 (relative to the SCP1 +1 TSS; the SCP1 TATA and mutTATA promoter backbones required different primers) were used to pair each barcode with its corresponding Inr variant. Reverse primers were selected from the NEBNext® Multiplex Oligos for Illumina® kits (New England Biolabs), which match the Illumina Read2-primer sequence present on the HARPE plasmid and corresponding cDNA. NGS PCR amplicons were size-selected on native 6% (w/v) polyacrylamide gels prior to Illumina sequencing.

HARPE sequencing and data processing

Illumina sequencing of NGS PCR amplicons was performed on NovaSeq 6000 or NovaSeq X Plus sequencing systems at the IGM Genomics Center, University of California, San Diego (supported by NIH SIG grant #S10 OD026929). NGS data processing was performed as described in Vo ngoc et al. (2020, 2023). Paired-end (PE100) sequences were required to have a perfect match to the 10-nt sequences immediately upstream and downstream of the randomized Inr region, which was required to be exactly 10 nt or 14 nt (for experiments with the 10-nt or 14-nt randomized Inr libraries, respectively). Sequences were also required to have a perfect match to the 10-nt sequences immediately upstream and downstream of the randomized barcode region, which was required to be exactly 15 nt or 17 nt (for experiments with the 10-nt or 14-nt randomized Inr libraries, respectively). All reads containing a match to the selection pattern were deemed usable and trimmed for sequences outside the randomized region. Conversion tables from barcode to Inr variant were built by paired-end sequencing of amplicons from the starting plasmid libraries. Both read 1 and read 2 were required to pass the above

screening criteria. Barcodes that were associated with multiple Inr variants were discarded (this was rare given the complexity of the 15-nt and 17-nt regions used). DNA and RNA datasets for all Inr HARPE experiments were matched to their corresponding barcode-to-Inr conversion tables. For the DNA datasets, we only used sequences with a minimum absolute read count of 10 and a minimum relative count of 0.2 or 0.75 reads per million (RPM; for experiments with the 10-nt or 14-nt randomized Inr libraries, respectively) so that low-confidence variants would not be included in the analyses. RNA dataset sequences were then matched to their corresponding DNA dataset. The transcription strength for each Inr sequence variant was defined as the RNA read count (in RPMs) divided by the DNA read count (in RPMs). Inr variants associated with multiple barcodes were combined, and their transcription strengths were computed as the average transcription strength across multiple barcodes.

SVR model training and optimization

Machine learning analyses were performed as described in Vo ngoc et al. (2020, 2023), except that we used functions from the Python package scikit-learn (version 1.3.0; Pedregosa et al. 2011) instead of the R package e1071 (version 1.7-2). For SVR training, we used the default radial basis function (RBF) kernel, which yielded the best results among those tested. Nucleotide variables for HARPE variants were computed as four categories (A, C, G, T) using one-hot encoding. These categories were used as the input features, and transcription strength was used as the output variable. For each SVR model, we set aside approximately 7,000-8,000 independent test sequences that represent the full range of transcription strengths. With the remaining sequences, we trained the SVR models with the 100,000 most active (best) variants and 100,000 randomly selected non-best variants. Grid search was performed for the hyperparameters cost and gamma, and cross validation was carried out with two independent test sets (two halves of the test sequences) that were not used for training (Supplemental Fig. S3). The selected hyperparameters for each model are as follows: for the Inr10 (with DPR) SVRb, Inr10 (with TATA) SVRb, Inr14 (with DPR) SVRb, and Inr14 (Inr only) SVRb models, we

used cost = 10 and gamma = 0.1; for the Inr14 (with TATA) SVRb, Inr14 (with DPR) SVRc, and Inr14 (with TATA) SVRc models, we used cost = 1 and gamma = 0.1.

Use of the SVR models

We used SVR models of the human Inr (this study), human TATA box ("SVRTATA"; Vo ngoc et al. 2020; GSE139635), human DPR ("SVRb"; Vo ngoc et al. 2020; GSE139635, which is the same as "SVRh"; Vo ngoc et al. 2023; GSE225570) and *Drosophila* DPR ("SVRd", Vo ngoc et al. 2023; GSE225570). The SVR models of the human TATA box, human DPR, and *Drosophila* DPR were trained by using the R package e1071. To facilitate the use of these models, we previously provided an R script, SVRpredict.R, which requires R with packages e1071 and docopt (Vo ngoc et al. 2023; GSE225570). In this work, SVR models of the human Inr were instead trained by using the Python package scikit-learn. To facilitate their use, we now provide SVRpredict.py, a Python script analogous to SVRpredict.R. SVRpredict.py requires Python with packages scikit-learn (version 1.3.0 strongly recommended), pandas, numpy, and joblib. Both SVR prediction scripts require the input of an SVR model file and a query sequence file (containing a column of sequences; the default column name is "sequence"), and they output a new file with predicted transcription strengths in an added column ("SVR_score"). Additional information may be included in the query sequence file, but the columns must be tab-delimited, and the column containing the sequences must be specified. Although the two scripts function similarly, SVRpredict.R must be used with the models of the TATA box and DPR (Vo ngoc et al. 2020, 2023), and SVRpredict.py must be used with the models of the Inr (this study).

Motif discovery

Motif discovery was performed with Hypergeometric Optimization of Motif EnRichment (HOMER; Heinz et al. 2010). findMotifs.pl was used to search for de novo motifs among the most active (best) HARPE sequence variants. Randomly selected non-best sequence variants

were used as background. WebLogo 3 (Crooks et al. 2004) was used to generate all sequence logos.

Identification of transcription start sites

Human transcription start sites (TSSs) were identified by using the GRO-cap method in GM12878 cells (Core et al. 2014; GSE60456) and csRNA-seq method in K562 cells (Duttke et al. 2019; GSE135498). *Drosophila* TSSs were identified by using the csRNA-seq method in *Drosophila* embryos (Delos Santos et al. 2022; GSE203135) and 5'-GRO-seq method in S2 cells (GSE68677). Focused TSSs in the human and *Drosophila* TSS data were identified as described in Vo ngoc et al. (2017) by using the Focus_TSS.py peak-calling algorithm. Minimum Focus Index (FI) thresholds ranging from 0.67 to 0.80 were used to define “focused” TSSs. The corresponding promoter sequences were extracted from the human genome [hg19 (GM12878) and hg38 (K562)] and *Drosophila* genome [dm6 (embryos) and dm3 (S2)] by using homerTools extract (Heinz et al. 2010).

Gene ontology analyses

Human transcription start sites (TSSs) from the GM12878 GRO-cap dataset (Core et al. 2014) were annotated by using annotatePeaks.pl from the HOMER suite (Heinz et al. 2010) with the hg19 genome assembly and GENCODE Release 19 annotations. GRO-cap TSSs located >200 nt from the nearest annotated TSS were excluded. The genes for which the GRO-cap TSSs are ≤200 nt from the nearest annotated TSSs were used as the “universe” (background) gene list. Gene ontology (GO) enrichment analyses were performed by using the R package clusterProfiler (Wu et al. 2021), with gene-to-GO term mappings provided by the org.Hs.eg.db annotation package. Ensembl gene IDs and the Biological Process (BP) ontology were used. Over-representation analysis (ORA) was used to identify GO terms enriched among genes whose corresponding promoters contained either a TATA box only (TATA+, Inr−, DPR−) or an Inr and DPR (TATA−, Inr+, DPR+). The activity of each element was evaluated by using the

SVR models of the human TATA box (Vo ngoc et al. 2020; GSE139635), human Inr [Inr10 (with DPR) SVRb model; this study], and human DPR (Vo ngoc et al. 2020; GSE139635). Approximately 4% of the “universe” genes contained a TATA box only (TATA+, Inr−, DPR−), and approximately 13% had an Inr and DPR (TATA−, Inr+, DPR+). ORA yielded 29 significant (adjusted $p < 0.05$) GO terms for TATA-only promoters and 0 (zero) significant GO terms for Inr+DPR promoters. Gene set enrichment analysis (GSEA) was also performed to identify GO terms with a skewed distribution across the full gene list ranked by TATA SVR score. This analysis identified 170 significant (adjusted $p < 0.05$) GO terms. The top 25 GO terms from the ORA and GSEA analyses are shown in Supplemental Table 4A and 4B, respectively.

Human TSSs from the K562 csRNA-seq dataset (Duttke et al. 2019) were annotated by using annotatePeaks.pl from the HOMER suite (Heinz et al. 2010) with the hg38 genome assembly and GENCODE Release 49 annotations. csRNA-seq TSSs located >200 nt from the nearest annotated TSS were excluded. For promoters containing a perfect match to the TCT motif (YC_{+1}TYTYY), the associated genes are listed in Supplemental Tables 3A and 3B, ranked by Inr (with DPR) and Inr (with TATA) SVRc scores, respectively.

Data analysis, statistics, and graphical displays

All general data processing and visualizations were performed in R (version 4.4.0) with R packages tidyr (v1.3.1), dplyr (v1.1.4), ggplot2 (v3.5.1), stringr (v1.5.1), Biostrings (v2.72.1), or in Python (version 3.12.1) with Python packages numpy (v1.26.4), pandas (v2.2.3), scipy (v1.12.0), matplotlib (v3.10.0), seaborn (v0.13.2), and biopython (v1.83).

Supplemental References

Core LJ, Martins AL, Danko CG, Waters CT, Siepel A, Lis JT. 2014. Analysis of nascent RNA identifies a unified architecture of initiation regions at mammalian promoters and enhancers. *Nat Genet* **46:** 1311–1320.

Crooks GE, Hon G, Chandonia JM, Brenner SE. 2004. WebLogo: A sequence logo generator. *Genome Res* **14:** 1188–1190.

Delos Santos NP, Duttke S, Heinz S, Benner C. 2022. MEPP: more transparent motif enrichment by profiling positional correlations. *NAR Genom Bioinform* **4:** lqac075

Dignam JD, Lebovitz RM, Roeder RG. 1983. Accurate transcription initiation by RNA polymerase II in a soluble extract from isolated mammalian nuclei. *Nucleic Acids Res* **11**: 1475–1489.

Duttke SH, Chang MW, Heinz S, Benner C. 2019. Identification and dynamic quantification of regulatory elements using total RNA. *Genome Res* **29:** 1836–1846.

Heinz S, Benner C, Spann N, Bertolino E, et al. 2010. Simple combinations of lineage-determining transcription factors prime cis-regulatory elements required for macrophage and B cell identities. *Mol Cell* **38:** 576–589.

Pedregosa F, Varoquaux G, Gramfort A, Michel V, Thirion B, Grisel O, Blondel M, Prettenhofer P, Weiss R, Dubourg V, et al. 2011. Scikit-learn: machine learning in Python. *J Mach Learn Res* **12:** 2825–2830.

Vo ngoc L, Cassidy CJ, Huang CY, Duttke SHC, Kadonaga JT. 2017. The human initiator is a distinct and abundant element that is precisely positioned in focused core promoters. *Genes Dev* **31:** 6–11.

Vo ngoc L, Huang CY, Cassidy CJ, Medrano C, Kadonaga JT. 2020. Identification of the human DPR core promoter element using machine learning. *Nature* **585:** 459–463.

Vo Ngoc, L, Rhyne, TE, Kadonaga, JT. 2023. Analysis of the *Drosophila* and human DPR elements reveals a distinct human variant whose specificity can be enhanced by machine learning. *Genes Dev* **37:** 377–382.

Wu T, Hu E, Xu S, Chen M, Guo P, Dai Z, Feng T, Zhou L, Tang W, Zhan L, et al. 2021. clusterProfiler 4.0: a universal enrichment tool for interpreting omics data. *Innovation (Camb)* **2:** 100141.
